# Lead fractionation and availability in contaminated soils: a comparative study of organic wastes and zeolite as stabilizing agents

**DOI:** 10.1007/s10653-026-03373-8

**Published:** 2026-08-05

**Authors:** Priscila Soares de Resende, Gordon W. Price, Rafael da Silva Teixeira, Isabela C. Filardi Vasques

**Affiliations:** 1 Department of Soils, Federal University of Viçosa, Viçosa, Minas Gerais 36570-900 Brazil; 2 Faculty of Agriculture, Dalhousie University, 21 Cox Rd, PO Box 550, Truro, NS B2N 5E3 Canada

**Keywords:** Lead, Organic amendments, Zeolite, Sequential extraction, Soil remediation, Availability

## Abstract

Lead (Pb) contamination in soils is a major environmental concern due to its persistence, toxicity, and bioaccumulation. This study evaluated the effects of organic amendments (sewage sludge, cattle manure, and poultry manure) and zeolite with different particle sizes (< 0.4, 2–8, and > 8 mm) applied at 1, 5, and 10% (v/v) on Pb mobility and availability in soil contaminated with Pb-rich mine tailings. Soil pH and acid-labile Pb extracted with Mehlich-1 were monitored for 30 days, and Pb fractions were assessed by sequential extraction at the end of the experiment. Mine tailings increased soil pH from 5.5 to 7.3 and stabilized acid-labile Pb at 223 mg kg^−1^ by day 8. Organic amendments altered pH and Pb availability, with stabilization occurring within 5–15 days depending on amendment type and dose. Poultry manure increased soil pH and reduced acid-labile Pb by 17 and 32% at 5 and 10%, respectively, whereas sewage sludge caused initial acidification followed by stabilization near neutral pH. All organic amendments promoted Pb redistribution toward more stable organic matter– and oxide-associated fractions. Zeolite significantly reduced acid-labile Pb across all particle sizes, with the fine fraction (< 0.4 mm) at 10% achieving the greatest immobilization (61%). Sequential extraction indicated Pb stabilization through increased association with crystalline Fe/Al oxides. Overall, poultry manure and fine zeolite effectively immobilized Pb, highlighting their potential as low-cost amendments for mitigating Pb contamination in mining-impacted soils.

**Supplementary Information:**

The online version contains supplementary material available at 10.1007/s10653-026-03373-8.

## Introduction

Lead (Pb) is a non-essential heavy metal (HM) occurring naturally in soils and is classified among the most hazardous elements to human health. It exists in soils at variable concentrations as a result of geochemical weathering of rocks and volcanic emissions (Mishra et al., 2025). Human activities have greatly intensified Pb enrichment in soils, particularly in urban and agricultural areas, where major sources include industrial effluents, waste disposal, construction debris, vehicular emissions, mining and smelting operations, and the extensive use of agrochemicals (Ankush et al., 2023). Its long-term persistence (>1000 years) in the environment, non-degradable nature, high toxicity, and ability to accumulate in the food chain pose significant risks to ecosystems and human health (Zhang & Wang, 2020). For instance, agricultural soils with high concentrations of Pb can lead to crop uptake, while in urban and industrial soils direct contact, dust inhalation, and water contamination cause adverse effects on gastrointestinal, renal, respiratory and nervous system of inhabitants of exposed communities (Mishra et al., 2025).

Globally, it is estimated that 500,000 ha are contaminated with harmful metal(oid)s at concentrations exceeding geochemical background or regulatory human toxicity thresholds (Azhar et al., 2022). In Brazil, the municipality of Boquira, located in the semi-arid northwest of Bahia State, represents one of the most emblematic cases of legacy Pb contamination associated with mining activities. From the 1950s until its closure in 1992, Boquira hosted one of the country’s largest Pb–Zn mines, where approximately 5 Mt of ore were extracted, generating large volumes of untreated mine tailings deposited in the vicinity of the urban area. More than three decades later, nearly 900 million liters of waste remain exposed without containment measures (Paes et al., 2025). This material has been eroded by water and wind, facilitating the dispersion of Pb-rich particles into nearby rural and urban areas (Paes 2023b; Paes et al., 2023a). As a result, soil contamination levels are elevated, posing significant risks to local populations and ecosystem function of neighbouring habitats.

Lead toxicity is related primarily to its bioavailability, whereby substantial fractions can be immobilized in soil matrices in forms that are not accessible for absorption by plants, microorganisms, or animals. Lead bioavailability is determined by its chemical speciation but and a complex interplay of other factors that regulate key soil processes, including adsorption, solubilization, precipitation, complexation, and chelation (Rajendran et al., 2022). These processes in turn are influenced by soil properties such as pH, mineralogy, clay content, organic matter (OM) content, and cation exchange capacity (CEC) (Zhu et al., 2019). Moreover, Pb bioavailability can also be influenced by metal affinity, presence of competing ions, organic exudates, plant and animal residues, microbial metabolites, and plant root activity (Zhu et al., 2019).

The mobility and bioavailability of Pb in soils can be mitigated through the application of organic amendments, including animal manures, composts, and biosolids, and inorganic materials such as natural zeolites. Organic amendments increase soil OM content acting to enhance Pb adsorption, thereby reducing its mobility; studies have demonstrated their effectiveness in immobilizing Pb and other HMs, particularly when nutrient-rich and low-metal manures are used (Khan et al., 2015; Venegas et al., 2015). This strategy may be transient, as changes in redox conditions or the presence of low-molecular-weight organic acids (e.g., citric and tartaric acids) can destabilize Pb–organic complexes, enhance Pb^2+^ desorption, and temporarily increase its bioavailability (Chen et al., 2024; Zhu et al., 2019). In addition, organic amendments may themselves also represent secondary sources of HMs, making their origin, treatment history, and metal content critical factors for safe application (Cele & Maboeta, 2016). On the other hand, zeolite, a microporous aluminosilicate mineral with crystalline framework, is widely used as natural adsorbents in water treatment and remediation of contaminated soils because it has strong adsorption of water, nutrients, and HMs. (Boostani et al., 2023; Doula et al., 2019; Papadopoulos et al., 2020; Zorpas et al., 2021).

Soil contamination is commonly assessed by measuring total metal concentrations; however, this metric alone is not a reliable indicator of environmental risk, as only a fraction of the total metal pool is mobile within the soil profile and available for uptake by plant roots (de Camargo et al., 2000). Typically, water-soluble and exchangeable forms are considered readily mobile and available to plants. Metals adsorbed in clay interlayers, bound to oxides or complexed with OM are considered less bioavailable (Sposito et al., 1982). To quantify these different forms of Pb in the soil sequential chemical extraction techniques are used (Kummer et al., 2011; Shuman, 1979; Sposito et al., 1982; Tessier et al., 1979). In combination with information on potential sources of HMs, this technique provides capacity to determine environmental distribution, bioavailability, and transport dynamics of metals in natural environments (Tessier et al., 1979). In this context, understanding the mechanisms regulating Pb mobility and bioavailability is essential for developing effective strategies to remediate contaminated soils and mitigating the environmental risks associated with HMs. The objective of this study was to evaluate the effects of differing organic amendments and zeolite on Pb availability and mobility in contaminated soils through sequential chemical extractions.

## Materials and methods

### Sampling and preparation of soil mixed with mine tailings

 Subsoil samples corresponding to the most representative expression of the Bw diagnostic horizon were collected from an Oxisol according to USDA Soil Taxonomy (Soil Survey Staff, 2022), classified as *Latossolo Vermelho-Amarelo* in the Brazilian Soil Classification System (SiBCS) (Santos et al., 2025), in Viçosa, Minas Gerais State, Brazil (-20.76159, -42.87469). This soil is one of the most widespread soil order in Brazil, covering approximately 31% of the country’s land area (Santos et al., 2011).

The clay fraction of the *Latossolo Vermelho-Amarelo* is predominantly composed of kaolinite, gibbsite, and goethite, reflecting the advanced stage of weathering of the gneiss-derived parent material (CBCS, 1995). This mineralogical assemblage is characteristic of highly weathered tropical soils and is associated with low natural fertility and a predominance of variable-charge surfaces. Among these minerals, goethite and gibbsite are particularly important due to their high specific surface area and abundance of reactive hydroxyl groups, which provide numerous sorption sites for metal ions (Xiang et al., 2025). It is important to note that these Fe and Al oxides possess relatively high points of zero charge (PZC), so their surfaces are commonly positively charged under the acidic conditions typical of tropical soils, favoring the adsorption of anions. Nevertheless, metal cations such as Pb may also be strongly retained through the formation of specific inner-sphere surface complexes with hydroxyl groups on oxide surfaces (Zhu et al., 2019). In addition, Fe and Al oxides can promote the formation of ternary surface complexes involving Pb and inorganic or organic ligands, thereby increasing Pb retention (Zhu et al., 2014). The formation of these complexes has been attributed to the greater affinity of metal–ligand species for oxide surfaces relative to free metal ions, making them more effective at overcoming electrostatic barriers during sorption onto positively charged minerals such as goethite (Ostergren et al., 2000). Although kaolinite generally exhibits lower surface reactivity than Fe and Al oxides (Potter & Yong, 1999), it may also contribute to Pb retention through cation exchange and surface complexation reactions. Therefore, the predominance of kaolinite, gibbsite, and goethite suggests a soil matrix capable of effectively immobilizing Pb through a combination of specific adsorption and surface complexation processes.

This soil presented high Al saturation (m = 69%), low base saturation (V = 1.1%), and very low exchangeable Ca^2+^ and Mg^2+^ concentrations (0.01 cmol_c_ dm^−3^), so it was incubated with CaCO_3_ and MgCO_3_ for two weeks to increase the base saturation to 70% as common practice in highly weathered tropical soils (Teixeira, 2017). After the incubation, the soil was mixed with Pb-rich mine tailing (up to 5,000 mg kg^−1^) from Boquira, Bahia State, Brazil (12º48′50″ S e 42º42′32″ W) at a proportion sufficient to achieve a final Pb concentration of 1000 mg kg^−1^ in the soil-tailings mixture (2023b; Paes et al., 2023a). This concentration was selected based on the investigation threshold of 180 mg kg^−1^ established for agricultural soils in Minas Gerais State, Brazil (Copam, 2011), as well as on previous studies from the region that reported Pb concentrations ranging from 300 to 22,468 mg kg^−1^ in surrounding soils (2023b; Paes, 2023a). Therefore, the selected concentration represents a realistic contamination scenario for soils affected by mining activities in Boquira and exceeds regulatory thresholds by a substantial margin, indicating a potential risk to human health and justifying the evaluation of Pb stabilization and availability in contaminated soils.

### Soil characterization

To fully characterize the soil used in the experiment, chemical and particle-size analyses were performed on air-dried fine earth (< 2 mm) according to the Brazilian Agricultural Research Corporation (Teixeira et al., 2017) (Table 1). Soil pH was determined in water using a 1:2.5 ratio (soil:solution). Available P, K, Fe, and Pb were extracted using Mehlich-1 solution (Mehlich, 1953), while S was extracted with monocalcium phosphate in acetic acid. Exchangeable cations (Ca^2+^, Mg^2+^, and Al^3+^) were extracted with 1 mol L^−1^ KCl. Potential acidity (H + Al) was determined using 0.5 mol L^−1^ calcium acetate buffered at pH 7.0. Organic matter content was estimated from organic carbon determined by the Walkley–Black method using the conversion factor 1.724 (Walkley & Black, 1934). Total N was determined by the Kjeldahl method. Particle-size distribution was determined by the pipette method using clay dispersed in water. Effective CEC was calculated as the sum of exchangeable bases (Ca^2+^, Mg^2+^, and K^+^) and exchangeable acidity (Al^3+^), whereas CEC at pH 7.0 was calculated as the sum of exchangeable bases and potential acidity (H + Al).

**Table 1:** Chemical composition and granulometric size distribution of the soil used in this study

Attributes	
pH in water	5.2
Organic matter (%)	0.6
Available P (mg dm^−3^)	0.0
Available K (mg dm^−3^)	3.0
Available S (mg dm^−3^)	10.7
Exchangeable Ca^2+^ (cmol_c_ dm^−3^)	0.01
Exchangeable Mg^2+^ (cmol_c_ dm^−3^)	0.01
Exchangeable Al^3+^ (cmol_c_ dm^−3^)	0.06
Available Fe (mg dm^−3^)	15.7
Total N (g kg^−1^)	0.4
Acid-labile Pb (mg dm^−3^)	0.25
Effective cation exchange capacity (cmol_c_ dm^−3^)	0.09
Cation exchange capacity at pH 7.0 (cmol_c_ dm^−3^)	2.73
H + Al (cmol_c_ dm^−3^)	2.7
Clay content (%)	80
Silt content (%)	0.8
Sand content (%)	19
Texture	clayey

### Sampling of soil amendments

The organic amendments chosen due to their lower cost and availability to local communities included poultry manure (PM), cattle manure (CM) and sewage sludge (SS). Manures were provided by Federal University of Viçosa, Department of Veterinary (Viçosa, Minas Gerais, Brazil), the SS was provided by the anaerobic sewage treatment plant (Viçosa, Minas Gerais, Brazil) and the zeolites were provided by Celta Brasil Company in three different particle size ranges: fine zeolite (< 0.4 mm), medium zeolite (2–8 mm) and coarse zeolite (> 8 mm).

### Mine tailings and soil amendments characterization

Macro and micronutrients and potential toxic elements were determined according to the EPA 3051A method (USEPA, 2007). Three grams of samples were ground with an agate mortar and sieved to 0.15 mm (150 mesh). Then, separately, aliquots of 0.5 g of mine tailings; 0.5 g of zeolite or 0.1 g of organic amendments were digested with a mixture of concentrated acids (HNO_3_ and HCl at 9:3 ratio). The PTE concentrations were determined by analyzing the filtered supernatant using Inductively Coupled Plasma–Optical Emission Spectroscopy (ICP-OES) (PerkinElmer Optima 8300). Results of the characterization are presented in Table 2.

**Table 2:** Chemical composition of the mine tailing, organic amendments and zeolite used in this study

Attributes	Mine tailing	Poultry manure	Cattle manure	Sewage sludge	Zeolite
pH in water	9.6	9.9	8.5	4.1	7.4
C-org (g kg^−1^)	11.9	312.90	291.70	274.80	-
Total N (g kg^−1^)	0.00	17.10	12.10	9.40	0.60
Total P (mg kg^−1^)	205.2	34,626.6	2235.6	3405.6	179.1
Total K (mg kg^−1^)	829.4	34,471.3	6333.4	764.5	12,489.1
Total S (mg kg^−1^)	2753.4	7654.6	2459.8	3235.7	632.6
Total Ca (mg kg^−1^)	10,130.1	154,007.3	8567.6	5864.9	15,996.1
Total Mg (mg kg^−1^)	5272.2	6766.3	2351.2	629.80	3370.9
Total Fe (mg kg^−1^)	56,799.9	2067.8	20,469.8	28,339.4	10,497.9
Total Al (mg kg^−1^)	2038.9	758.4	9277.4	11,675.78	25,042.3
Total Pb (mg kg^−1^)	5161.2	< 0.04	< 0.04	28.48	< 0.04
Total Cd (mg kg^−1^)	40.4	0.00	0.00	0.00	0.00
Total Cr (mg kg^−1^)	7.4	3.6	35.4	29.1	0.00
Total Ni (mg kg^−1^)	9.7	< 0.02	< 0.02	< 0.02	< 0.02

Previous mineralogical studies have identified galena (PbS), anglesite (PbSO₄), cerussite (PbCO₃), and hydrocerussite [Pb_3_(CO_3_)_2_(OH)_2_] as the main Pb-bearing phases present in the mine tailings (Lima & Bernardez., 2023; Paes et al., 2025).

### Experimental setup

Two independent and simultaneous soil incubation experiments were conducted in the Geochemistry Laboratory of the Department of Soil Science at the Federal University of Viçosa over a one-month period using a double factorial design (3 × 3 + 1), comprising three soil amendments (either organic or inorganic) applied at three application rates and an unamended soil mixed with Pb-rich mine tailing used as a control (CK) (Table 3). Each treatment was established with three replicates, resulting in a total of 30 experimental units for each expriment. Amendments were applied on a volumetric basis; therefore, the corresponding mass-based application rates varied according to amendment bulk density. Based on typical dry bulk densities reported for PM, CM, SS, and zeolite, the highest application rate (10% v/v) corresponded approximately to 90, 110, 140, and 200 g kg^−1^, respectively. Fifteen kilograms of the mixture soil + mine tailing were distributed among several 500 mL plastic pots and randomly assigned to the experimental treatments in a bench-scale setup. All pots were maintained under controlled temperature conditions (25–30 °C). Field capacity was estimated using the soil moisture equivalent method described by Ruiz et al. (2003), and soil moisture was maintained at approximately 80% of field capacity throughout the incubation period (Paes et al., 2023b; Paes et al., 2023a). Every 48 h, the pots were weighed to determine water loss, and deionized water was added to restore the target moisture content. After water addition, the contents of each pot were transferred to individual plastic bags and thoroughly homogenized to ensure uniform moisture distribution before being returned to the corresponding pots. Maintaining a stable moisture content and periodically homogenizing the samples ensured consistent contact among Pb, the soil matrix, and the organic amendments, and facilitating the establishment of sorption–desorption equilibrium during the incubation period. These conditions were maintained to provide a controlled environment for evaluating Pb stabilization by the organic amendments.

**Table 3:** Treatment identification and corresponding experimental conditions

Organic amendment	Application rate	Treatment ID	Inorganic amendment	Application rate	Treatment ID
unamended	0%	CK	unamended	0%	CK
sewage sludge	1% (v/v)	SS 1%	fine zeolite	1% (v/v)	Z < 0.4 mm 1%
sewage sludge	5% (v/v)	SS 5%	fine zeolite	5% (v/v)	Z < 0.4 mm 5%
sewage sludge	10% (v/v)	SS 10%	fine zeolite	10% (v/v)	Z < 0.4 mm 10%
cattle manure	1% (v/v)	CM 1%	medium zeolite	1% (v/v)	Z 2-8 mm 1%
cattle manure	5% (v/v)	CM 5%	medium zeolite	5% (v/v)	Z 2-8 mm 5%
cattle manure	10% (v/v)	CM 10%	medium zeolite	10% (v/v)	Z 2-8 mm 10%
poultry manure	1% (v/v)	PM 1%	coarse zeolite	1% (v/v)	Z > 8 mm 1%
poultry manure	5% (v/v)	PM 5%	coarse zeolite	5% (v/v)	Z > 8 mm 5%
poultry manure	10% (v/v)	PM 10%	coarse zeolite	10% (v/v)	Z > 8 mm 10%

### Acid-labile Pb and pH stabilization time

Because Pb occurs in soils in multiple chemical forms and no single extractant is universally accepted as a standard predictor of its bioavailability or mobility, various extraction methods have been used in the literature, including CaCl₂, DTPA, Mehlich-1, and Mehlich-3 (Araújo et al., 2022; da Silva et al., 2022; Tavares & Oliveira, 2017). These extractants target different geochemical pools of Pb through distinct mechanisms such as ion exchange, chelation, and acid dissolution, often resulting in different estimates of metal availability.

During the incubation period, Mehlich-1 extractions were performed to quantify acid-labile Pb fraction. Mehlich-1 is a dilute double-acid extractant (0.05 mol L^−1^ HCl + 0.0125 mol L^−1^ H_2_SO_4_) that primarily targets acid-labile forms of metals in soil and is commonly used as an operational indicator of potentially mobile metal pools rather than a direct measure of bioavailability (da Silva et al., 2022). Therefore, the extracted Pb was interpreted as a proxy for Pb availability and mobility in the soil. For clarity, this fraction is hereafter referred to as acid-labile Pb.

The temporal stabilization of soil pH and acid-labile Pb was evaluated by collecting soil samples from the upper layer of each pot before water reposition on days 1, 3, 5, 8, 15, 21, and 30. Samples were collected using a volumetric soil scoop calibrated for approximately 3 g of soil, and the exact sample mass was checked by weighing. Soil, mine tailings, PM, CM, and SS were air-dried and passed through a 2 mm sieve before the experimental set up. Zeolite was used without sieving because particle size was one of the experimental variables evaluated in the study. Soil pH was measured prior to chemical extraction in the same samples. The resulting extracts were centrifuged, and Pb concentrations in the supernatants were determined by flame atomic absorption spectrometry (FAAS) (AA240FS, Agilent Technologies®, USA).

### Pb sequential extraction

On day 30, soil samples were collected from each pot, homogenized, and oven-dried for subsequent analyses. Soil sample masses were recorded using a precision balance with four decimal places to obtain a pre-extraction dry sample mass and post-extraction final sample mass. Samples were dried in the oven at 40°C for 12 hours between extractions. After extraction, the residues were washed using 0.5 mol L^-1^ (NH_4_)_2_CO_3_ solution and deionized water (except for the soluble fraction) to remove excess salts, followed by centrifugation at 3,500 rpm for 10 minutes. The fractions from each extraction were stored in polyethylene bottles previouly cleaned with 10% (v/v) HNO3 solution and ultrapure water and placed in a refrigerator at 4°C. In this study we followed the method described by Kummer et al., (2011), with a modification in the determination of the residual fraction (F7), for which Pb concentrations were quantified using the EPA 3051 digestion method to obtain pseudo-total concentrations (USEPA, 2007). Kummer et al., (2011) applied this procedure to evaluate Pb and Zn distribution in eight tropical soil profiles directly influenced by Pb mining and smelting activities.

Lead contents were fractionated as follows: soluble (ultrapure water, Milli-Q) (F1), exchangeable (Ca(NO_3_)_2_, 0.5 mol L^−1^) (F2); bound to carbonates (NaAc 1.0 mol L^−1^ at pH 5.0) (F3); bound to OM (NaClO 0.7 mol L^−1^) (F4); bound to amorphous Fe and Al oxides (ammonium oxalate 0.2 mol L^−1^ + oxalic acid 0.2 mol L^−1^ + ascorbic acid 0.1 mol L^−1^) (F5); bound to crystalline Al oxides and 1:1 and 2:1 minerals (NaOH 1.25 mol L^−1^) (F6); and residual (conc. HNO_3_ + conc. HCl) (F7). The supernatants were collected for Pb analysis using FAAS (AA240FS, Agilent Technologies®, USA).

### Statistical analysis

The experiment was conducted in a completely randomized design comprising ten treatments with three replicates per treatment, resulting in a total of 30 experimental units for each experiment: organic amendments and zeolite. Statistical analysis were conducted separated for each experiment. Prior to conducting parametric analyses, all variables were subjected to the Lilliefors test to verify normality and to Cochran’s test to assess homogeneity of variances. These preliminary tests ensured compliance with the assumptions of independence, normality, and homoscedasticity required for analysis of variance (ANOVA) (Zhou et al., 2023).

For variables that did not initially meet these assumptions, data were subjected to appropriate transformations (e.g., logarithmic or square-root) and re-evaluated until acceptable distributions were achieved. Once assumptions were met, ANOVA was applied. Statistical significance was assessed at α = 0.05 (95% confidence level).

To evaluate temporal dynamics, contrasts with Bonferroni correction were used to control the family-wise error rate. These contrasts compared consecutive sampling times (Day 30 vs. Day 21; Day 21 vs. Day 15; Day 15 vs. Day 8; Day 8 vs. Day 5; Day 5 vs. Day 3; and Day 3 vs. Day 1). Within each organic amendment and zeolite particle size treatment, means were compared using Tukey’s HSD test at a 5% significance level.

## Results

### Acid-labile Pb and pH stabilization time in the soil spiked with the mine tailing

**Fig. 1:**
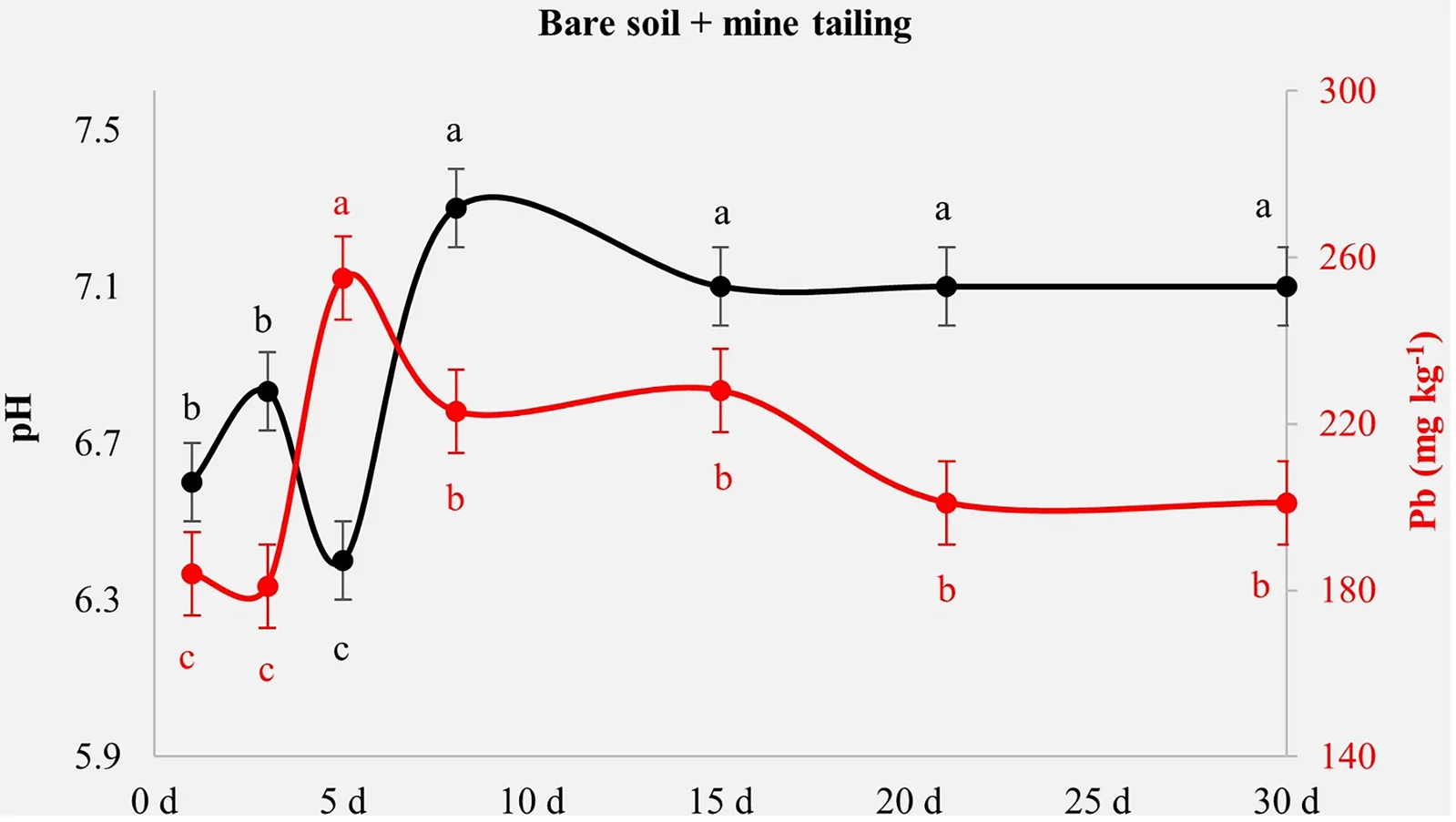
Variation in soil pH and acid-labile Pb concentration over a 30-day incubation with Pb contaminated mine tailings. Values are means ± standard error, n = 6. Comparisons were performed between consecutive sampling times (Day 3 vs. Day 1; Day 5 vs. Day 3; Day 8 vs. Day 5; Day 15 vs. Day 8; Day 21 vs. Day 15; and Day 30 vs. Day 21). Different lowercase letters between consecutive samples indicate significant differences based on Holm-Bonferroni adjusted contrasts to control the family-wise error (*p* < 0.05, *n* = 6)

Soil pH and acid-labile Pb extracted with Mehlich-1 measured over a 30-day period are shown in Fig. 1. Contamination of the bare soil (pH 5.5) with mine tailings (pH 9.6) increased soil pH to 6.6 within the first 24 h and to 7.3 by day 8, with no significant differences (*p*  >*0.05*) detected at later sampling intervals (day 8 vs. day 15; day 15 vs. day 21; day 21 vs. day 30). Similarly, acid-labile Pb stabilized after eight days at 223 mg kg^−1^, with no significant differences observed in subsequent measurements.

### pH and acid-labile Pb dynamics in soils with organic amendments

**Fig. 2:**
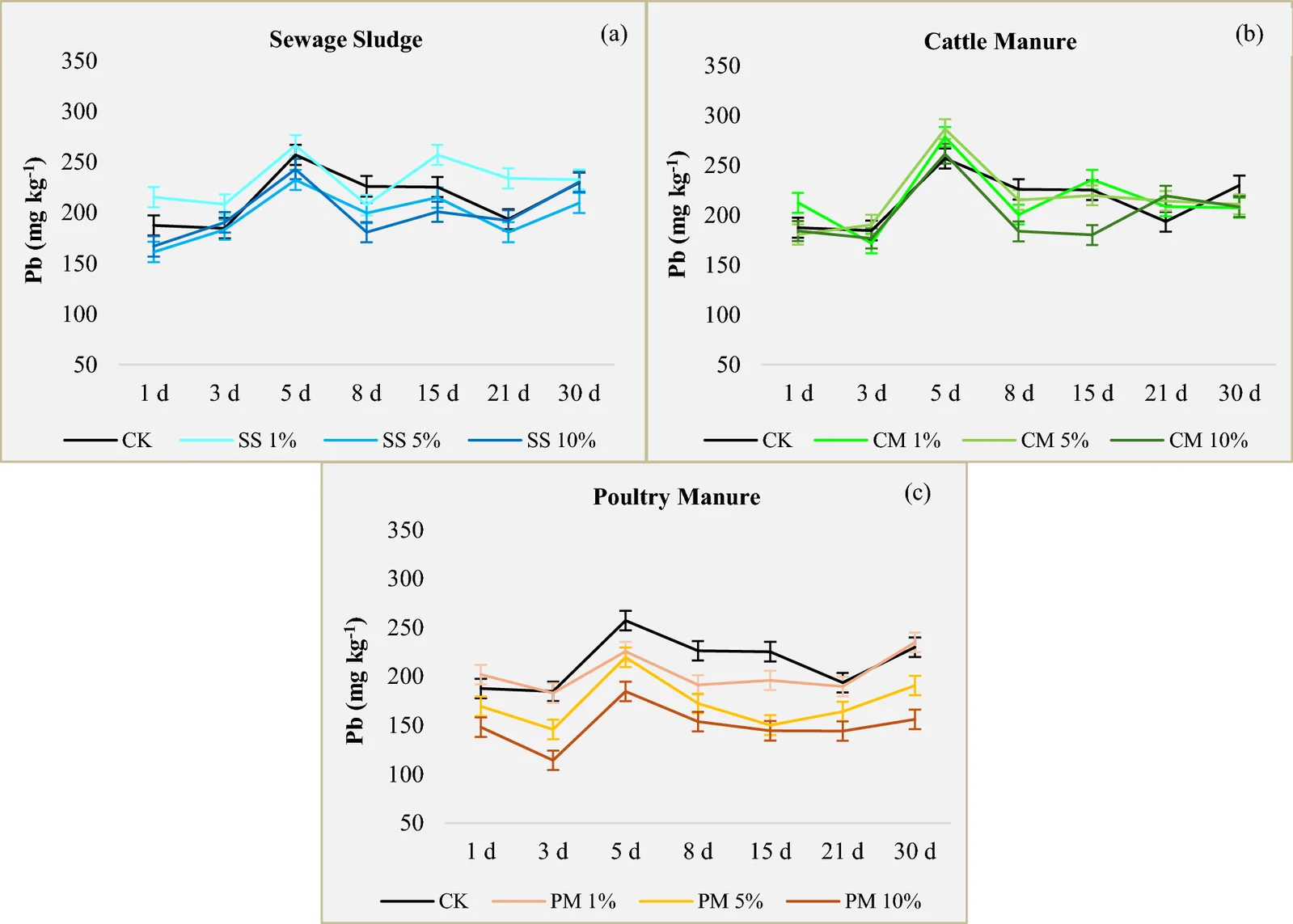
Variation in acid-labile Pb concentration over a 30-day incubation with organic amendments applied to soil at 1%, 5%, and 10% (v/v): **a** sewage sludge (SS); **b** cattle manure (CM); and **c** poultry manure (PM), compared with the control (CK; bare soil + mine tailing). Values represent means ± standard error (n = 3)

When organic amendments were incorporated into soils, the resulting dynamics of soil pH and acid-labile Pb were altered, as shown in Fig. S1a–c and Fig. 2a–c, respectively, and the results were summarized in Tables S1, S2 and S3. In SS treatments (Fig. S1a and 2a), the 5% and 10% application rates caused a significant decrease in pH within the first 24 h. However, by Day 8, values had already stabilized, with no further differences observed between consecutive sampling times (*p* > 0.05). By Day 21 pH levels were similar to those of the CK. In contrast, the 1% SS treatment did not significantly differ from CK throughout the incubation period. Acidic-labile Pb, responded differently based on SS application rates. In the SS1% treatment, acid-labile Pb concentrations stabilized by Day 15, whereas in SS5% stabilization occurred earlier, by Day 5. The SS10% treatment continued to show fluctuations up to the last sampling, with significant differences (p<0.05) still observed between Day 30 and Day 21. Overall, all three SS treatments followed a similar trend to the CK, with two exceptions: (i) SS1% showed significantly higher acid-labile Pb than CK at Days 15 and 21, and (ii) SS10% showed a significant decrease relative to CK at Day 8. By Day 30, however, all SS treatments converged to Pb concentrations comparable to CK.

In CM treatments, pH values stabilized rapidly, with no significant differences from the CK observed after Day 8 (*p* > *0.05*) (Fig. S1b). Acid-labile Pb showed a similar trend: by Day 8, no further differences were detected between consecutive sampling times at any application rate (Fig. 2b). By Day 21, all CM treatments exhibited acid-labile Pb concentrations statistically comparable to CK.

In PM treatments (Figs. S1c and 2c), soil pH was consistently higher than the CK during the first five days across all application rates, and the magnitude of this increase was concentration-dependent, with the 10% PM treatment exhibiting the greatest rise. By Day 8, however, pH values in all PM treatments converged to levels comparable to CK. In parallel, acid-labile Pb concentrations were reduced relative to CK in the PM5% (− 17%) and PM10% (− 32%) treatments, whereas PM1% followed a pattern similar to CK. Notably, while acid-labile Pb concentrations stabilized across treatments by Day 8, PM was the only organic amendment in which this stabilization occurred at values consistently lower than CK, and these reductions were maintained thereafter.

### Lead fractions across different organic amendments in soil

**Fig. 3:**
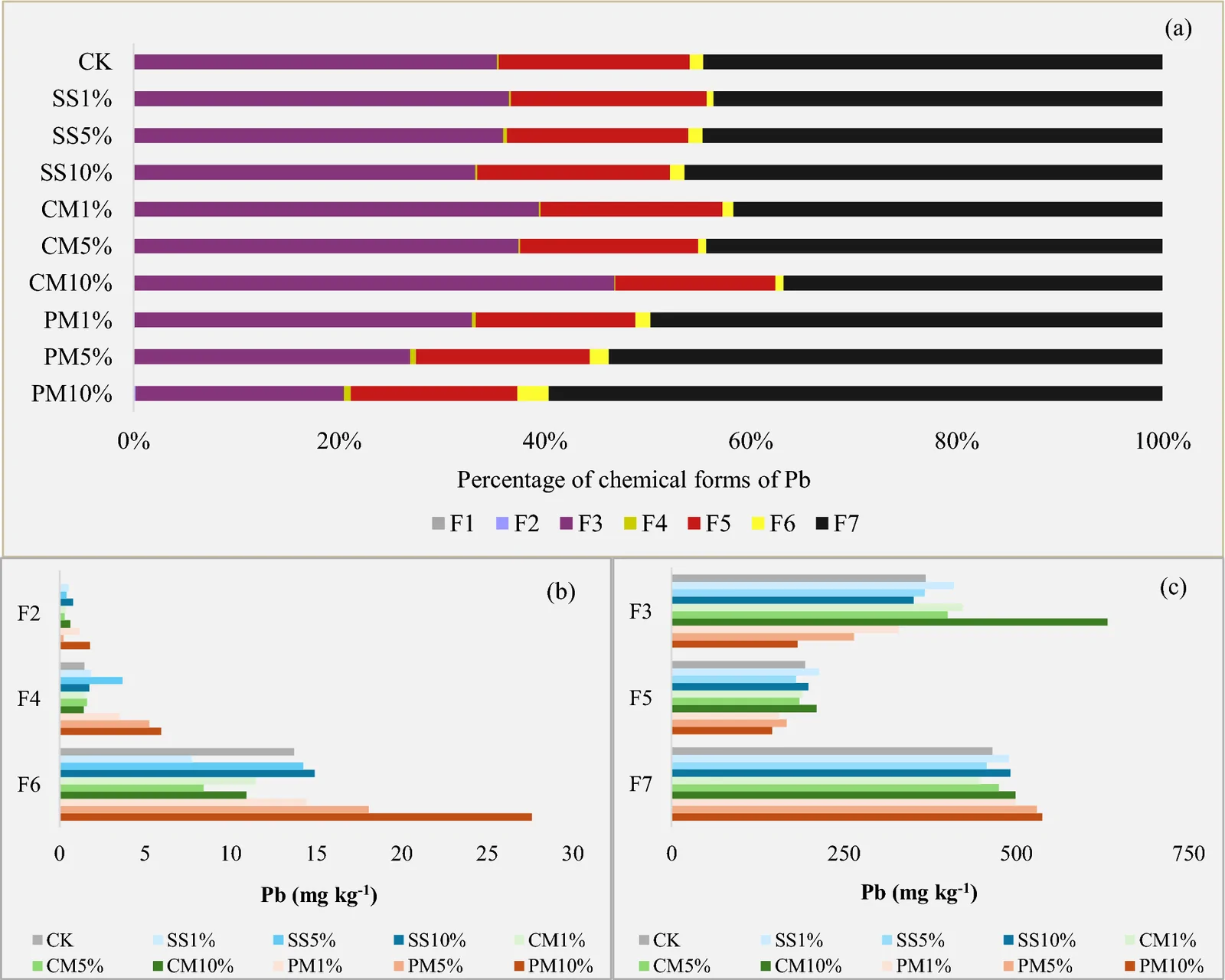
Percentage of Pb chemical forms (**a**) and Pb distribution among sequential extraction fractions (**b, c**) after 30 days of incubation in bare soil, mine tailings, and soils amended with organic amendments (sewage sludge—SS; cattle manure—CM; and poultry manure—PM) applied at 1%, 5%, and 10% (v/v). Values are means ± standard error (n = 3). Sequential extraction fractions are water soluble (F1), exchangeable (F2), carbonates (F3), organic matter (F4), amorphous Fe oxide (F5), crystalline Fe/Al oxides and 1:1 minerals (F6) and residual (F7). F1 and F2 are present but not visually distinguishable due to their very low contribution. CK represents the control (unamended soil)

The distribution of Pb fractions and after the 30-day incubation are presented in Fig. 3a–c. The water-soluble fraction (F1) was negligible across all treatments. The exchangeable fraction (F2), representing the most labile Pb pool, ranged from 0.0 to 1.8 mg kg^−1^. Despite its low absolute contribution, all organic amendments significantly increased Pb in F2 relative to CK (0.0 mg kg^−1^). Among treatments, SS and CM did not differ, whereas PM1% (1.2 mg kg^−1^) and PM10% (1.8 mg kg^−1^) showed comparable values that were significantly higher than PM5% (0.2 mg kg^−1^). Notably, PM1% and PM10% exhibited the greatest enrichment in exchangeable Pb among all amendments. The carbonate-bound fraction (F3) constituted the largest Pb pool, ranging from 183 to 561 mg kg^−1^. Compared with CK (369 mg kg^−1^), CM10% (633 mg kg^−1^) increased Pb concentration by 72%, whereas PM5% (265 mg kg^−1^) and PM10% (183 mg kg^−1^) reduced Pb concentration by 28% and 50%, respectively. Among treatments, CM10% contained significantly more Pb than PM5% and PM10% while no difference was observed between the two PM treatments. Pb bound to OM (F4) was comparatively low (1.4–5.9 mg kg^−1^); however, several treatments significantly increased this pool relative to CK (1.4 mg kg^−1^), including SS5% (+ 157%: 3.7 mg kg^−1^), PM1% (+ 144%: 3.5 mg kg^−1^), PM5% (+ 265%: 5.2 mg kg^−1^), and PM10% (+ 314%: 5.9 mg kg^−1^). Among treatments, SS5% and PM1% did not differ, whereas both were significantly lower than PM5% and PM10%, which were statistically similar to each other. For the amorphous Fe/Al oxide-bound fraction (F5; 145–213 mg kg^−1^), a significant reduction was observed only under PM10% (− 25%: 145 mg kg^−1^) compared to CK (194 mg kg^−1^). Relative to CK (13.7 mg kg^−1^), the crystalline Al oxide and minerals 1:1-bound fraction (F6; 7.7–27.6 mg kg^−1^) decreased Pb concentration in SS1% (− 44%: 7.7 mg kg^−1^) and CM5% (− 39%: 8.4 mg kg^−1^), whereas PM10% (27.6 mg kg^−1^) substantially increased this pool by + 101%. Among treatments, SS1% and CM5% were statistically similar, and both contained significantly less Pb than PM10%. Finally, the residual fraction (F7; 447–538 mg kg^−1^) did not differ significantly among treatments and CK (465 mg kg^−1^), indicating that the most recalcitrant Pb pool remained unaffected by organic amendments.

### pH and acid-labile Pb dynamics in soils with zeolite at different particle sizes

**Fig. 4:**
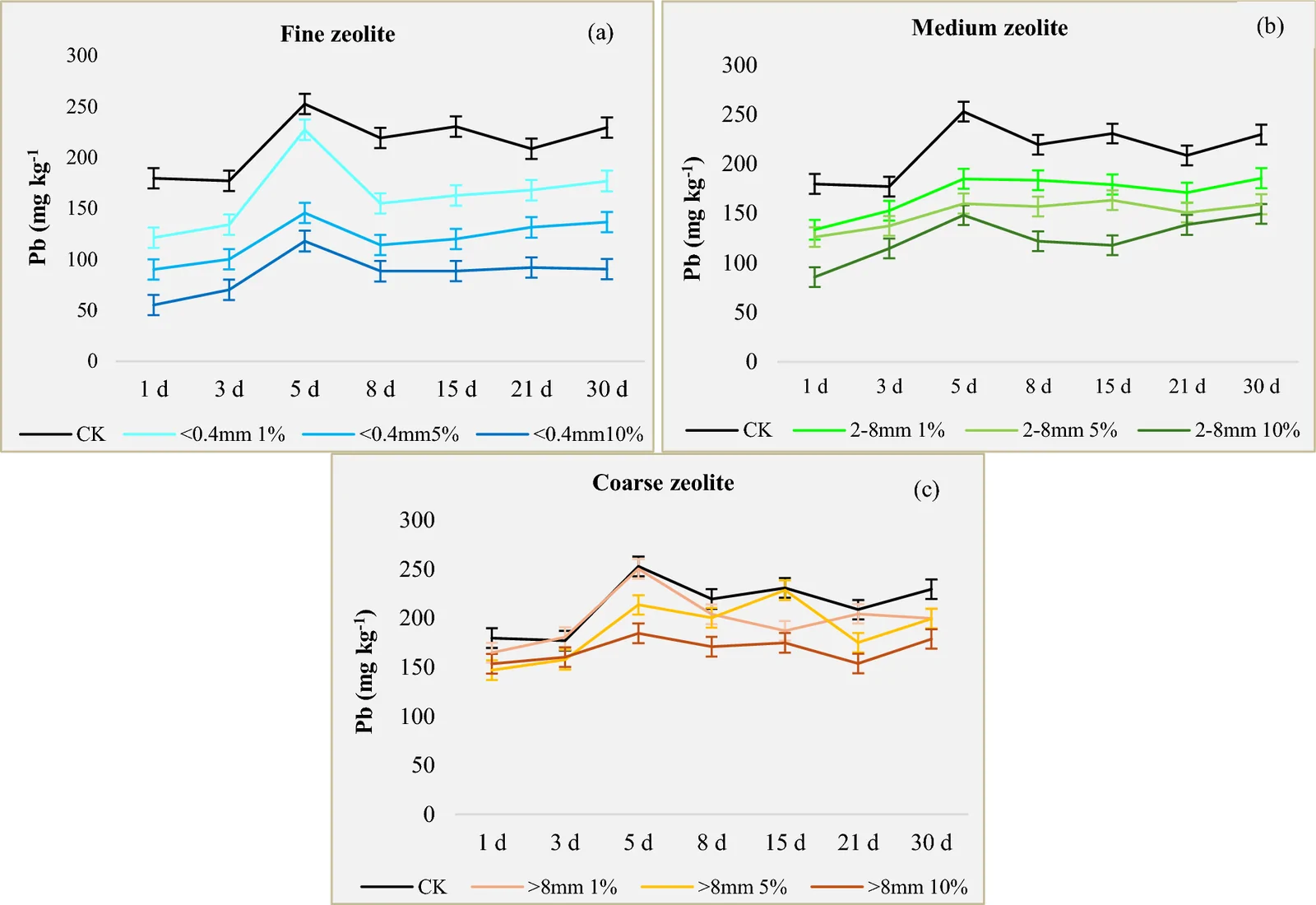
Variation in acid-labile Pb concentration over a 30-day incubation with zeolite of different particle sizes applied at 1%, 5%, and 10% (v/v): **a** < 0.4 mm; **b** 2–8 mm; **c** > 8 mm), compared with the control (CK; bare soil + mine tailing). Values represent means ± standard error (n = 3)

In the treatment with 1% application of fine zeolite (< 0.4 mm), soil pH showed no significant variation across the sequential sampling times, indicating a relatively stable behavior throughout the 30-day incubation (Fig. S2a). At the 5% and 10% application rates, pH exhibited progressive adjustments in the early days and reached stabilization by day 8. All zeolite treatments (1%, 5%, and 10%) converged to values comparable to CK, around neutral pH (7.1). Acid-labile Pb concentrations stabilized by day 8 across all application rates, indicating rapid immobilization in response to zeolite amendment regardless of the application rate (Fig. 4a). From day 1, all zeolite treatments exhibited consistently lower Pb concentrations than CK, indicating an immediate immobilization effect. A clear dose-dependent effect was evident, with higher application rates producing progressively greater reductions in acidic-labile Pb. By day 30, acid-labile Pb concentrations were 229 mg kg^−1^ in CK, compared to 177 mg kg^−1^, 137 mg kg^−1^, and 90 mg kg^−1^ for the < 0.4 mm treatments at 1%, 5%, and 10%, respectively, with the 10% amendment exhibiting the most substantial immobilization (Table S4).

All application rates of medium-sized zeolite (2–8 mm) exhibited a pH pattern similar to CK, stabilizing around neutral values by day 8 (Fig. S2b). Acid-labile Pb concentrations also reached equilibrium by day 8 across all application rates, indicating rapid Pb immobilization independent of the application rate (Fig. 4b). The stabilization time for both measured variables followed the same pattern as the fine zeolites. All treatments consistently maintained lower acid-labile Pb concentrations than CK (229 mg kg^−1^) throughout the incubation, confirming the immobilization effect of medium-sized zeolite, although at lower magnitude than observed for the fine zeolite (1%: 185 mg kg^−1^; 5%: 159 mg kg^−1^; 10%: 149 mg kg^−1^). In contrast to the fine zeolite, no significant differences were detected among the 1%, 5%, and 10% treatments by day 21, suggesting that within this particle size range, increasing zeolite dosage did not substantially enhance Pb immobilization beyond the effect achieved at the lowest rate (Table S5).

For the coarse zeolite treatments (> 8 mm), no significant differences in pH were observed between consecutive samplings across the 1%, 5%, and 10% application rates beyond day 8, consistent with the patterns observed for the fine and medium particle fractions (Fig. S2c). When compared with CK, all treatments exhibited similar neutral pH values as CK by day 8, with the exception of the highest application rate that stabilized at higher pH values after day 15 (CK: 7.1 and > 8 mm10%: 7.4). Acid-labile Pb concentrations in the coarse zeolite treatments stabilized earlier than in the finer and medium zeolites, reaching equilibrium by day 5 (Fig. 4c). A transient fluctuation was observed in the 5% treatment at day 21, but overall trends remained stable. Compared with CK, the 10% application consistently resulted in lower acid-labile Pb concentrations, although the magnitude of reduction was smaller than in the fine particles and comparable to that of the medium particles. The 1% and 5% treatments exhibited occasional fluctuations but generally showed values similar to CK, converging by day 30 to levels equivalent to the 10% treatment. At day 30, acid-labile Pb concentrations were 229 mg kg^−1^ in CK, 199 mg kg^−1^ in both the 1% and 5% treatments, and 179 mg kg^−1^ in the 10% treatment (Table S6).

### Lead fractions across different application rates and sizes of zeolite in soil

**Fig. 5:**
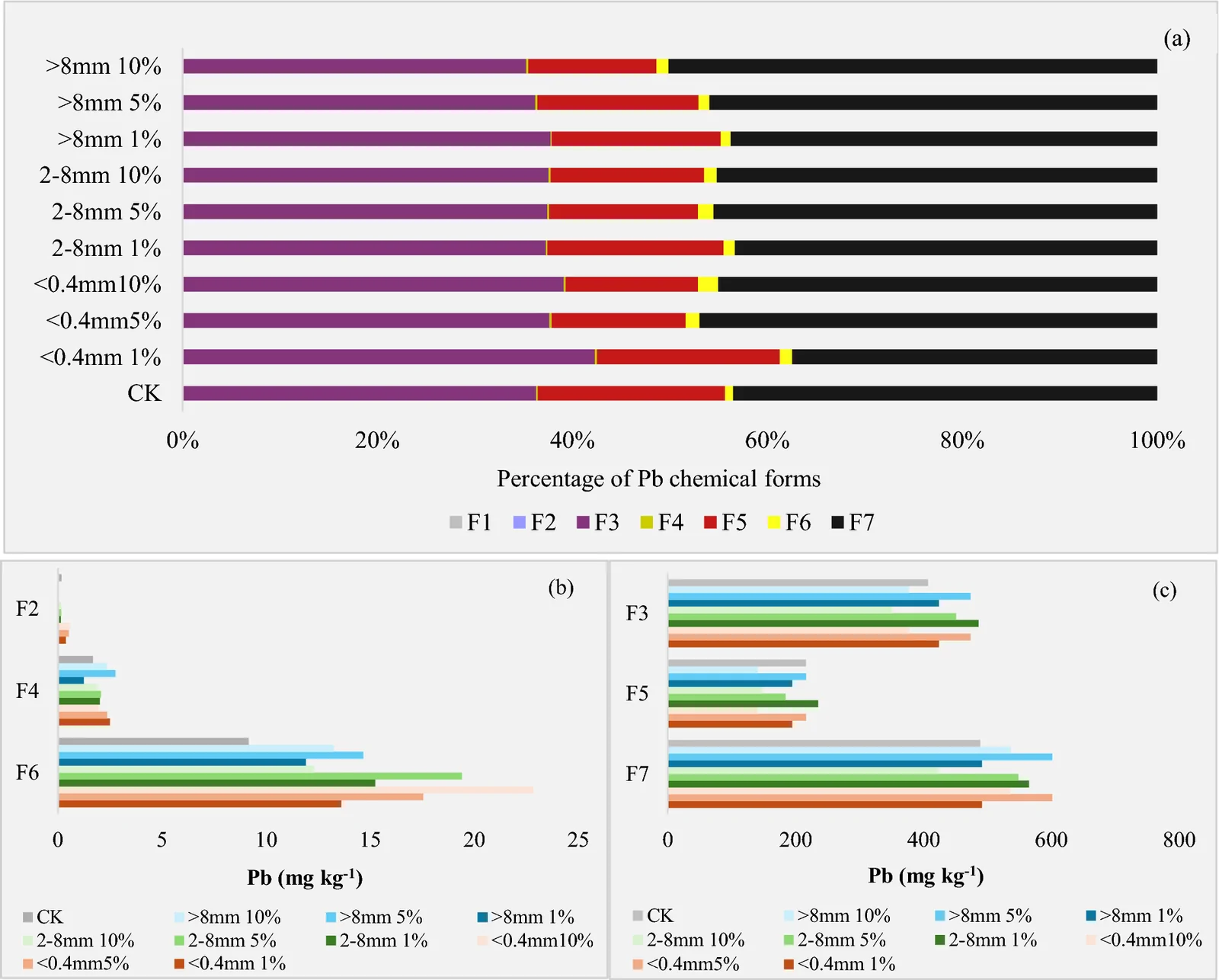
Percentage of Pb chemical forms (**a**) and Pb distribution among sequential extraction fractions (**b, c**) after 30 days of incubation in bare soil, mine tailings, and soils amended with zeolite of different particle sizes (> 8 mm, 2–8 mm and < 0.4 mm) applied at 1%, 5%, and 10% (v/v). Values are means ± standard error (n = 3). Sequential extraction fractions are water soluble (F1), exchangeable (F2), carbonates (F3), organic matter (F4), amorphous Fe oxide (F5), crystalline Fe/Al oxides and 1:1 minerals (F6) and residual (F7). F1 and F2 are present but not visually distinguishable due to their very low contribution. CK represents the control (unamended soil)

The fractions of Pb after 30 days of incubation with zeolite in soils are presented in Fig. 5a–c. The water-soluble fraction (F1) remained negligible across all treatments. The exchangeable fraction (F2; 0.0–0.6 mg kg^−1^) was slightly enhanced by fine zeolite, with increases of + 135% (0.4 mg kg^−1^), + 218% (0.5 mg kg^−1^) and + 243% (0.6 mg kg^−1^) at 1%, 5% and 10%, respectively, compared to CK (0.16 mg kg^−1^). No significant differences were observed among fine zeolite treatments. The carbonate-bound fraction (F3; 352–487 mg kg^−1^) was the largest pool, with no significant differences comparing treatments and CK (407 mg kg^−1^). Pb bound to OM (F4; 1.4–2.8 mg kg^−1^) showed a significant increase only under > 8 mm zeolite at 5% (+ 64%: 2.8 mg kg^−1^) compared to CK (1.4 mg kg^−1^). The amorphous Fe oxide-bound fraction (F5; 140–235 mg kg^−1^) decreased under higher zeolite applications, with reductions of − 31% for < 0.4 mm 10% (149 mg kg^−1^), − 32% for 2–8 mm 10% (148 mg kg^−1^), and − 35% for > 8 mm 10% (141 mg kg^−1^) compared to CK (216 mg kg^−1^). No differences were found among these three treatments. The crystalline Fe/Al oxide-bound fraction (F6; 9–23 mg kg^−1^) increased in several treatments compared to CK (9 mg kg^−1^), particularly with fine and medium zeolite particles, including < 0.4 mm 5% (+ 92%: 18 mg kg^−1^) and 10% (+ 150%: 23 mg kg^−1^), 2–8 mm at 1% (+ 67%: 15 mg kg^−1^) and 5% (+ 112%: 19 mg kg^−1^), and > 8 mm at 5% (+ 60%: 15 mg kg^−1^). No significant differences were observed among these five treatments. Finally, the residual fraction (F7; 413–601 mg kg^−1^) was unaffected, with no differences detected across treatments compared to CK (489 mg kg^−1^).

## Discussion

### Effects of Pb-rich mine tailings contamination on soil pH and acid-labile Pb

Soil physical and chemical properties, particularly pH, play a central role in controlling Pb mobility and bioavailability (Rahman et al., 2024). Under acidic conditions, Pb remains predominantly in soluble forms, whereas increasing pH promotes adsorption, complexation, and precipitation reactions that reduce its mobility and potential plant uptake (Kumari et al., 2023; Zulfiqar et al., 2019).

Our results demonstrate that soil pH and acid-labile Pb followed similar stabilization dynamics after contamination with mine tailings. Soil pH increased rapidly within the first 24 h due to the high carbonate content of the mine tailings, as later confirmed by sequential extraction, but only stabilized around neutral values after approximately  one week. This behavior is attributed to the high carbonate content of the mine tailings, which is associated with the presence of carbonate-bearing Pb minerals identified in previous mineralogical studies of the same material (Lima and Bernadez, 2023; Paes et al., 2025). These phases contribute to the buffering capacity of the system, promoting neutral to slightly alkaline conditions.

Acid-labile Pb concentration required a similar equilibration period, stabilizing after approximately eight days. This delayed response likely reflects the gradual redistribution of metals among soil fractions through sorption–desorption equilibria, precipitation–dissolution reactions, and interactions with organic and mineral surfaces (Zulfiqar et al., 2019). The close correspondence between pH and Pb stabilization indicates that Pb availability was strongly influenced by the same geochemical processes controlling soil acidity.

These findings highlight the importance of allowing an adequate equilibration period before evaluating remediation treatments in contaminated soils. Failure to account for these stabilization processes may result in misinterpretation of amendment performance and metal availability, particularly in short-term adsorption and ecotoxicological studies where incubation periods shorter than one week are still commonly employed (Alexandrino et al., 2021; Ding et al., 2023; Nguyen-Phuong et al., 2024; Singh et al., 2024).

### Effects of organic amendments and zeolite with different particle sizes on soil pH

The application of SS initially reduced soil pH, whereas CM and PM increased it. The acidification observed in SS treatments was mainly associated with its lower intrinsic pH (4.1), compared with the soil (5.5) and the mine tailings (9.6). In contrast, the alkaline nature of CM (pH 8.5) and PM (pH 9.9) increased soil pH, particularly in PM treatments. Alkaline amendments play a key role in mitigating the toxicity of cationic HMs in soils by promoting their adsorption onto negatively charged surface sites and promoting precipitation reactions (Bolan et al., 2014; Kumari et al., 2023).

Despite these contrasting initial responses, soil pH in all amended treatments converged to values similar to the CK (pH 7.1) within approximately one week. This stabilization likely reflects the strong buffering capacity of the soil–mine tailing system, resulting from the interaction between alkaline mine tailings and clay-rich soil (80% of clay). Over time, transient reactions associated with OM decomposition (e.g., release of organic acids, ammonification, and carbonate dissolution) and exchangeable cations redistribution approached equilibrium, leading to stable near-neutral pH values (Rahman et al., 2023; Sindhu et al., 2022). The high organic carbon content of all amendments may also have contributed to the buffering through increased CEC (Walker & Bernal, 2008).

Zeolite had little influence on soil pH. Although the zeolite was slightly alkaline (pH 7.4), its aluminosilicate framework is relatively inert and does not function as a liming agent. Minor increases in pH following zeolite application have been reported only for more alkaline zeolites and less buffered soils (Zhou et al., 2020). In the present study, the strongly buffered soil–mine tailing matrix, characterized by abundant carbonate phases and high clay content, likely limited any measurable pH effect of zeolite, which induced only transient pH shifts during the initial stages of application.

### Effects of organic amendments and zeolite with different particle sizes on soil acid-labile Pb

Organic amendments and zeolites can reduce metal mobility through several mechanisms, including adsorption, complexation, precipitation, and ion-exchange (Zorpas et al., 2021). The relative contribution of each mechanism depends on the physical and chemical properties of the amendment and soil conditions, such as pH, mineralogy, texture and OM content. In the present study, however, these individual mechanisms were not directly distinguished. Therefore, the observed changes in acid-labile Pb should be interpreted as the combined effect of the different stabilization processes promoted by each amendment.

Acid-labile Pb concentrations required approximately one week to stabilize across all treatments in our study, following a temporal pattern similar to soil pH. This synchronicity suggests that Pb partitioning between the soil solution and solid phase was influenced, at least in part, by the same buffering processes governing pH. Because Pb availability generally decreases as pH increases, fluctuations in acidity and alkalinity during the initial days likely affected the balance between dissolved Pb^2+^ and Pb retained through adsorption or precipitation reactions. Once pH stabilized, Pb concentrations also reached equilibrium (Porter et al., 2004).

The strong correspondence between soil pH and acid-labile Pb concentrations indicates that pH was an important factor controlling Pb mobility in the studied system. However, pH modification should not be considered independent of amendment effects, as changes in soil acidity are an inherent consequence of amendment application and would also occur under field conditions. Therefore, the observed reductions in Pb mobility reflect the combined influence of pH-induced precipitation and adsorption processes together with other amendment-mediated mechanisms, including complexation by organic functional groups, cation exchange, and interactions with mineral surfaces. Because the experimental design was not intended to isolate the relative contribution of these mechanisms, the results should be interpreted as the overall effect of the amendments on Pb stabilization rather than evidence of a single dominant process.

After stabilization, acid-labile Pb concentrations in soils amended with SS (1, 5 and 10%), CM (1, 5 and 10%), and PM1% were similar to the CK (~ 230 mg Pb kg^−1^) indicating limited Pb immobilization. Similar results have been reported for manure-amended mine tailing, where Pb extractability remained unchanged relative to the control (Zhou et al., 2025). In contrast, other studies have reported reductions in metal availability following the application of manure or compost (Clemente et al., 2006; Walker et al., 2003), highlighting the influence of soil properties, amendment characteristics, and experimental conditions on stabilization efficiency. In the present study, the rapid buffering of soil pH likely limited the long-term effect of these amendments on Pb availability. Although the initial acidification observed in SS treatments may have temporarily increased Pb mobilization during the early phase, this effect disappeared once soil–mine tailing system returned to near-neutral pH. Consequently, Pb availability remained essentially unchanged relative to the CK treatment after the equilibrium was established.

In contrast, PM applied at higher rates (PM5% and PM10%) reduced acid-labile Pb concentrations below CK levels, and these reductions persisted after stabilization. Several mechanisms likely explain this outcome: the combined influence of increased pH, complexation of Pb by organic functional groups, and precipitation of Pb-bearing minerals promoted by the high OM, calcium, and phosphorus contents of the amendment (Bolan et al., 2014; Kumari et al., 2023; Li et al., 2017; Liu et al., 2009; Seshadri et al., 2017; Walker et al., 2003). These mechanisms collectively reduced Pb mobility and contributed to the greater immobilization observed in PM treatments.

In contrast to the organic amendments, natural zeolite significantly reduced acid-labile Pb concentrations, with the greatest reduction (60.7%) observed for the finest particle size at the highest application rate. Similar reductions in Pb availability following zeolite application have been reported in previous studies (Boostani et al., 2023; Chang et al., 2019; Contin et al., 2019).

The high surface area, porosity, and CEC of zeolites promote Pb retention through adsorption and ion-exchange processes, while finer particles provide a larger reactive surface area for metal immobilization (Li et al., 2023). These findings suggest that, unlike organic amendments, zeolites acted primarily as a direct adsorbent and was therefore more effective at reducing the acid-labile Pb fraction in the soil. However, the present study was conducted under controlled incubation conditions over a relatively short period (30 days). Additional studies are needed to evaluate the long-term stability of Pb immobilization under field conditions, where fluctuations in pH, redox potential, moisture, and competing ions may affect the persistence of metal retention by zeolite. Furthermore, although acid-labile Pb was substantially reduced, this extraction method represents only one operationally defined fraction and does not directly assess plant uptake or ecological risk.

### Effects of organic amendments and zeolite with different particle sizes on Pb fractions

Sequential extraction provides a more comprehensive assessment of metal mobility than total metal concentrations by partitioning metals among fractions with contrasting stability and potential bioavailability. In the present study, Pb was predominantly associated with the carbonate-bound fraction (F3: 20–44%) and the residual fraction (F7:37–60%), with a smaller contribution from amorphous phases (F5; 13–19%). This distribution reflects the high carbonate content of the Boquira mine tailings (Paes et al., 2025) and indicates that a large proportion of Pb was already associated with relatively stable pools. Overall, F3 acts as short-term sinks for Pb, and F5 and F7 provide long-term stabilization.

The exchangeable fraction (F2) exhibited contrasting responses depending on the extractant employed. When Ca(NO₃)₂ was used, all organic amendments and fine zeolite treatments increased F2 relative to the CK, with PM at 10% showing the highest values. However, despite the large relative increases, absolute concentrations remained low, reaching a maximum of only 1.8 mg kg^−1^. In contrast, Mehlich-1 extraction revealed substantial reduction in acidic-labile Pb in PM5%, PM10%, and particularly in the fine- and medium-sized zeolite treatments, with fine zeolite at 10% reducing acidic-labile Pb by approximately 60%.

These contrasting results highlight the importance of extractant selection when assessing metal availability. Neutral salt extractants such as Ca(NO₃)₂ and CaCl₂ primarily estimate the readily exchangeable pool by promoting cation exchange reactions without substantially altering soil pH or metal speciation (Menzies et al., 2007). In contrast, Mehlich-1, an acidic extractant composed of HCl and H₂SO₄, mobilizes metals through proton-promoted dissolution of carbonates and weakly bound mineral phases (Andrade & Sobral, 2016; Li & Shuman, 1997). Mehlich-3 (0.2 mol L^−1^ acetic acid, 0.25 mol L^−1^ ammonium nitrate, 0.013 mol L^−1^nitric acid, 0.015 m mol L^−1^ ammonium fluoride and 0.001 mol L^−1^ EDTA) presents an intermediate behavior, combining acidic dissolution with chelation and ion exchange, whereas DTPA is a chelating extractant designed to estimate potentially plant-available metals through complexation reactions (Li & Shuman, 1997; Mehlich, 1984; Tavares & Oliveira, 2017). Consequently, extractants commonly used to assess available metals may target distinct geochemical pools and therefore provide contrasting estimates of metal mobility. The inverse responses observed between Ca(NO_3_)_2_ and Mehlich-1 suggest that the amendments increased the amount of weakly exchangeable Pb (F2 from sequential extraction) while simultaneously decreasing the fraction susceptible to acid-induced mobilization (acid-labile extracted by Mehlich-1). Future studies should evaluate the relationship between these extractants and Pb uptake by plants to identify which methodology best predicts metal bioavailability and to support the development of standardized assessment protocols for contaminated soils.

The carbonate-bound pool (F3) was the second dominant fraction, consistent with the alkaline nature of the mine tailings (pH 9.6) and the occurrence of cerussite (PbCO_3_) and hydrocerussite [Pb_3_(CO_3_)_2_(OH)_2_] identified in these materials by Paes et al. (2025), indicating that a substantial portion of Pb was associated with carbonate phases. Zeolite treatments did not significantly affect F3, suggesting limited influence on carbonate equilibria. In contrast, CM10% increased the carbonate-bound fraction, a response similar to that reported by Gomes et al. (2022) following the application of organic amendments. Conversely, PM10% reduced F3, indicating a different stabilization pathway. Cao et al. (2002) observed a decrease in carbonate-bound Pb and a concomitant increase in more stable fractions after phosphate addition. Although the mechanisms responsible for Pb redistribution were not directly assessed in the present study, the high phosphorus content of PM may have promoted the transfer of Pb from carbonate-associated forms to less labile fractions.

The most pronounced effects of organic amendments on less labile fractions were observed under PM treatments. Poultry manure increased F4 at all application rates and promoted a shift from F5 to F6 at higher doses. Similar redistribution patterns have been reported for biosolids, biochar, and composted plant residues (Anastopoulos et al., 2019; Gomes et al., 2022). Lead has a strong affinity for OM, favoring its association with oxidizable fractions and reducing its mobility; metals associated with OM- and oxide-bound fractions are generally considered less available for plant uptake (Li et al., 2022; McBride, 1989). It should be emphasized that under specific conditions, such as during OM mineralization, metals bound to OM may be remobilized (Almås et al., 1999).

The increase in F6 accompanied by a decrease in F5 suggests progressive stabilization of Pb within more recalcitrant mineral-associated pools. This redistribution is particularly relevant in Oxisols, where advanced weathering favors the accumulation of crystalline Fe and Al minerals relative to amorphous phases. Iron oxides are important sinks for HMs because they retain Pb through surface complexation and surface precipitation, two major mechanisms controlling Pb sequestration in soils (Shi et al., 2021; Silveira, 2002). Although amorphous phases usually possess greater surface reactivity and metal adsorption capacity than crystalline minerals (Catalano et al., 2011), their relatively low abundance in highly weathered soils may limit their contribution to overall Pb retention. Consequently, the shift from F5 to F6 observed under PM and zeolite treatments likely represents the transfer of Pb from more reactive but less abundant amorphous phases to more stable crystalline mineral associations, which are less susceptible to dissolution and subsequent metal release. However, because metal retention by Fe-bearing phases may be affected by changes in redox conditions, alterations in soil redox potential could promote the dissolution of these phases and influence Pb remobilization (Marinho et al., 2019).

The residual fraction (F7) remained unaffected across all treatments, indicating that neither organic amendments nor zeolite mobilized Pb from geochemically stable mineral phases. Similar stability of the residual fraction has been reported in other studies involving organic amendments, whereas inorganic amendments such as phosphate and lime may increase Pb incorporation into residual forms (Gomes et al., 2022).

## Conclusions

This study evaluated the effectiveness of organic amendments (sewage sludge, cattle manure, and poultry manure) and zeolite with different particle sizes for reducing Pb mobility and availability in a soil contaminated with mine tailings. The results demonstrated that a stabilization period of at least one week is necessary in experiments with artificially contaminated soils to ensure equilibrium of soil pH and acid-labile Pb before assessing amendment performance.

Among the tested materials, poultry manure and zeolite were the most effective in reducing Pb mobility, whereas sewage sludge and cattle manure showed limited ability to decrease the acid-labile Pb concentration. Zeolite acted primarily through sorption processes without significantly altering soil pH, and its effectiveness increased with decreasing particle size and increasing application rate. The finest zeolite applied at 10% achieved the greatest reduction in acid-labile Pb, decreasing this fraction by approximately 60%.

Sequential extraction showed that organic amendments primarily redistributed Pb among exchangeable, carbonate-, OM-, and mineral-associated fractions without affecting the residual pool. Poultry manure promoted the transfer of Pb into less labile fractions, likely due to the combined effects of OM complexation and phosphorus-induced precipitation. Zeolite also altered Pb partitioning toward more stable forms, reinforcing its effectiveness as a stabilization amendment. Despite these changes, the residual fraction remained unaffected across all treatments, indicating that neither organic amendments nor zeolite mobilized Pb from geochemically stable mineral phases.

The contrasting responses obtained with Ca(NO_3_)_2_ and Mehlich-1 extractions highlight the importance of extractant selection when assessing Pb availability. While Ca(NO_3_)_2_ reflects readily exchangeable Pb, Mehlich-1 estimates a broader acid-labile pool, and the inverse responses observed between these extractants demonstrate that different methods may provide substantially different interpretations of metal mobility. These findings emphasize the need for greater standardization in the assessment of Pb availability in contaminated soils.

Overall, these results highlight the potential of zeolite, particularly fine-particle zeolite, as an effective amendment for reducing Pb mobility in mine tailing-contaminated soils. However, the highest immobilization efficiency was achieved at a relatively high amendment rate (10%), which may limit large-scale field application due to economic and operational constraints. Furthermore, both sequential extraction and chemical extractants provide operationally defined measures of Pb mobility and do not directly assess long-term stability or plant uptake. Future studies should therefore focus on optimizing amendment rates, evaluating stabilization under field conditions, and establishing relationships between plant Pb uptake and different chemical extractants (CaCl_2_, Ca(NO_3_)_2_, DTPA, Mehlich-1, and Mehlich-3) to support the development of standardized protocols for assessing remediation effectiveness.

## Supplementary Information

Below is the link to the electronic supplementary material.

Supplementary file1 (DOCX 90 KB)

## Data Availability

The data supporting this study’s findings are available from the corresponding author upon reasonable request.

## References

[CR1] Alexandrino, R. C. S., Lima, F. R. D., Martins, G. C., Natal-da-Luz, T., Sousa, J. P., Guilherme, L. R. G., & Marques, J. J. (2021). Lead acetate ecotoxicity in tropical soils. *Ecotoxicology,**30*(6), 1029–1042. 10.1007/s10646-021-02443-0 34191243 10.1007/s10646-021-02443-0

[CR2] Almås, Å., Singh, B. R., & Salbu, B. (1999). Mobility of cadmium-109 and zinc-65 in soil influenced by equilibration time, temperature, and organic matter. *Journal of Environmental Quality,**28*(6), 1742–1750. 10.2134/jeq1999.00472425002800060008x

[CR3] Anastopoulos, I., Massas, I., Pogka, E.-E., Chatzipavlidis, I., & Ehaliotis, C. (2019). Organic materials may greatly enhance Ni and Pb progressive immobilization into the oxidizable soil fraction, acting as providers of sorption sites and microbial substrates. *Geoderma,**353*, 482–492. 10.1016/j.geoderma.2019.06.035

[CR4] Andrade, M., and Sobral, L. F. (2016). Extrações com Mehlich-1, Mehlich-3 e DTPA em Argissolo fertilizado com zinco, manganês e cobre (Boletim de Pesquisa e Desenvolvimento, 124). Aracaju: Embrapa Tabuleiros Costeiros. https://www.infoteca.cnptia.embrapa.br/infoteca/handle/doc/1065210

[CR5] Ankush, Dhanda, A., Lamba, S., Jakhar, R., Diwedi, A., Kumar, S., & Singh, V. (2023). Source and distribution of lead in soil and plant: A review. In N. Kumar & A. K. Jha (Eds.), *Lead toxicity: Challenges and solutions. Environmental Science and Engineering Series *(pp. 3–16). Springer. 10.1007/978-3-031-37327-5_1

[CR6] Araújo, B. D., Maia, R., Arantes-Garcia, L., Oki, Y., Negreiros, D., de Assis, I. R., & Fernandes, G. W. (2022). Aftershocks of the Samarco disaster: Diminished growth and increased metal content of *Raphanus sativus* cultivated in soil with mining tailings. *Acta Scientiarum. Biological Sciences,**44*, e59175. 10.4025/actascibiolsci.v44i1.59175

[CR7] Azhar, U., Ahmad, H., Shafqat, H., Babar, M., Shahzad Munir, H. M., Sagir, M., Arif, M., Hassan, A., Rachmadona, N., Rajendran, S., Mubashir, M., & Khoo, K. S. (2022). Remediation technique for elimination of heavy metal pollutants from soil: A review. *Environmental Research,**214*, 113918. 10.1016/j.envres.2022.113918 35926577 10.1016/j.envres.2022.113918

[CR8] Bolan, N., Kunhikrishnan, A., Thangarajan, R., Kumpiene, J., Park, J., Makino, T., Kirkham, M. B., & Scheckel, K. (2014). Remediation of heavy metal(loid)-contaminated soils: To mobilize or to immobilize? *Journal of Hazardous Materials,**266*, 141–166. 10.1016/j.jhazmat.2013.12.018 24394669 10.1016/j.jhazmat.2013.12.018

[CR9] Boostani, H. R., Hardie, A. G., & Najafi-Ghiri, M. (2023). Lead stabilization in a polluted calcareous soil using cost-effective biochar and zeolite amendments after spinach cultivation. *Pedosphere,**33*(2), 321–330. 10.1016/j.pedsph.2022.06.052

[CR10] Cao, X., Ma, L. Q., Chen, M., Singh, S. P., & Harris, W. G. (2002). Impacts of phosphate amendments on lead biogeochemistry at a contaminated site. *Environmental Science & Technology,**36*(24), 5296–5304. 10.1021/es020697j 12521153 10.1021/es020697j

[CR11] Catalano, J. G., Luo, Y., & Otemuyiwa, B. (2011). Effect of aqueous Fe(II) on arsenate sorption on goethite and hematite. *Environmental Science & Technology,**45*(20), 8826–8833. 10.1021/es202445w 21899306 10.1021/es202445w

[CR12] Cele, E. N., & Maboeta, M. S. (2016). A greenhouse trial to investigate the ameliorative properties of biosolids and plants on physicochemical conditions of iron ore tailings: Implications for an iron ore mine site remediation. *Journal of Environmental Management,**165*, 167–174. 10.1016/j.jenvman.2015.09.029 26433357 10.1016/j.jenvman.2015.09.029

[CR13] Chang, J. H., Liu, Q. F., Yu, J., Wang, Y. T., Peng, W. D., Chen, J. Y., & Liu, W. (2019). Effect, immobilization, and cooperativity of amendments on remediation of Pb-contaminated soil. *Applied Ecology and Environmental Research,**17*(5), 10637–10654. 10.15666/aeer/1705_1063710654

[CR14] Chen, F., Zhang, W., Hua, Z., Zhu, Y., Jiang, F., Ma, J., & Gómez-Oliván, L. M. (2024). Unlocking the phytoremediation potential of organic acids: A study on alleviating lead toxicity in canola (*Brassica napus* L.). *Science of the Total Environment,**914*, 169980. 10.1016/j.scitotenv.2024.169980 38215837 10.1016/j.scitotenv.2024.169980

[CR15] Clemente, R., & Bernal, M. P. (2006). Fractionation of heavy metals and distribution of organic carbon in two contaminated soils amended with humic acids. *Chemosphere,**64*(8), 1264–1273. 10.1016/j.chemosphere.2005.12.058 16481023 10.1016/j.chemosphere.2005.12.058

[CR16] Contin, M., Miho, L., Pellegrini, E., Gjoka, F., & Shkurta, E. (2019). Effects of natural zeolites on ryegrass growth and bioavailability of Cd, Ni, Pb, and Zn in an Albanian contaminated soil. *Journal of Soils and Sediments,**19*, 4052–4062. 10.1007/s11368-019-02359-7

[CR17] COPAM - Conselho Estadual de Política Ambiental, 2011. Deliberação Normativa Conjunta COPAM/CERH n° 171, de 22 de dezembro de 2011. Publicação Diário do Executivo, Minas Gerais, 23/12/2011. Retrived July 27, 2026, from https://www.siam.mg.gov.br/sla/download.pdf?idNorma=20095

[CR18] da Silva, D. R., Schaefer, C. E. G. R., Kuki, K. N., Santos, M. F. S., Heringer, G., & da Silva, L. C. (2022). Why is *Brachiaria decumbens* Stapf. a common species in the mining tailings of the Fundão dam in Minas Gerais, Brazil? *Environmental Science and Pollution Research,**29*, 79168–79183. 10.1007/s11356-022-21345-0 35708810 10.1007/s11356-022-21345-0

[CR19] de Camargo, M. S., dos Anjos, A. R. M., Rossi, C., & Malavolta, E. (2000). Adubação fosfatada e metais pesados em Latossolo cultivado com arroz. *Scientia Agricola,**57*(3), 513–518. 10.1590/S0103-90162000000300022

[CR20] Ding, H., Liu, J., Li, Q., Liu, Z., Xia, K., Hu, L., Wu, X., & Yan, Q. (2023). Highly effective adsorption and passivation of Cd from wastewater and soil by MgO- and Fe₃O₄-loaded biochar nanocomposites. *Frontiers in Environmental Science,**11*, 1239842. 10.3389/fenvs.2023.1239842

[CR21] Doula, M. K., Zorpas, A. A., Inglezakis, V. J., Pedreño, J. N., & Bilalis, D. J. (2019). Optimization of heavy polluted soil from olive mill waste through the implementation of zeolites. *Environmental Engineering and Management Journal,**18*(6), 1297–1309. 10.30638/eemj.2019.124

[CR22] Gomes, F. P., Barreto, M. S. C., Amoozegar, A., & Alleoni, L. R. F. (2022). Immobilization of lead by amendments in a mine-waste impacted soil: Assessing Pb retention with desorption kinetics, sequential extraction, and XANES spectroscopy. *Science of the Total Environment,**807*(1), 150711. 10.1016/j.scitotenv.2021.150711 34626622 10.1016/j.scitotenv.2021.150711

[CR23] Khan, F., Khan, M. J., Samad, A., Noor, Y., Rashid, M., & Jan, B. (2015). In-situ stabilization of heavy metals in agriculture soils irrigated with untreated wastewater. *Journal of Geochemical Exploration,**159*, 1–7. 10.1016/j.gexplo.2015.07.002

[CR24] Kumari, U., Pankaj, Yadav, S., Jangra, P., Raj, D., & Bhardwaj, K. K. (2023). The dynamics of lead in plant–soil interactions. In N. Kumar & A. K. Jha (Eds.), *Lead toxicity: Challenges and solutions**Environmental Science and Engineering*. Springer, Switzerland AG. pp. 17–29 10.1007/978-3-031-37327-5_2

[CR25] Kummer, L., Melo, V., de, Barros, Y. J., & de Azevedo, J. C. R. (2011). Extrações sequenciais de chumbo e zinco em solos de área de mineração e metalurgia de metais pesados. *Revista Brasileira De Ciência Do Solo,**35*(6), 2005–2018. 10.1590/S0100-06832011000600017

[CR26] Li, Z., & Shuman, L. M. (1997). Mehlich-1- and DTPA-extractable lead in soils in relation to properties. *Communications in Soil Science and Plant Analysis,**28*(3–5), 351–363. 10.1080/00103629709369795

[CR27] Li, X., Meng, D., Li, J., Yin, H., Liu, H., Liu, X., Cheng, C., Xiao, Y., Liu, Z., & Yan, M. (2017). Response of soil microbial communities and microbial interactions to long-term heavy metal contamination. *Environmental Pollution,**231*(1), 908–917. 10.1016/j.envpol.2017.08.057 28886536 10.1016/j.envpol.2017.08.057

[CR28] Li, Y., Gao, B., Xu, D., Lu, J., Zhou, H., & Gao, L. (2022). Heavy metals in the water-level-fluctuation zone soil of the Three Gorges Reservoir, China: Remobilization and catchment-wide transportation. *Journal of Hydrology,**612*(1). 10.1016/j.jhydrol.2022.128108

[CR29] Li, Y., Giordano, A., Ajmone-Marsan, F., & Padoan, E. (2023). Bioaccessibility of Pb in health-related size fractions of contaminated soils amended with phosphate. *Science of the Total Environment,**855*, 158831. 10.1016/j.scitotenv.2022.158831 36165822 10.1016/j.scitotenv.2022.158831

[CR30] de Lima, L. R. P. A., & Bernardez, L. A. (2023). Mineralogical and geochemical characterization of the tailings from the former lead–zinc flotation plant of Boquira mine Brazil. *Environmental Earth Sciences,**82*, 10. 10.1007/s12665-022-10678-1

[CR31] Liu, L., Chen, H., Cai, P., Liang, W., & Huang, Q. (2009). Immobilization and phytotoxicity of Cd in contaminated soil amended with chicken manure compost. *Journal of Hazardous Materials,**163*(2–3), 563–567. 10.1016/j.jhazmat.2008.07.004 18692313 10.1016/j.jhazmat.2008.07.004

[CR32] Marinho, C. H., Giarratano, E., Domini, C. E., Garrido, M., & Gil, M. N. (2019). Potential mobility assessment of metals in salt marsh sediments from San Antonio Bay. *Environmental Monitoring and Assessment,**191*, 723. 10.1007/s10661-019-7895-0 31696305 10.1007/s10661-019-7895-0

[CR33] McBride, M. B. (1989). Reactions controlling heavy metal solubility in soils. In B. A. Stewart (Ed.), *Advances in soil science* (pp. 1–55). Springer. 10.1007/978-1-4613-8847-0_1

[CR34] Mehlich, A. (1953). Determination of P, Ca, Mg, K, Na and NH₄. Short test method used in soil testing division. Department of Agriculture, Raleigh, North Carolina. https://www.ncagr.gov/divisions/agronomic-services/mehlich-1953/download?attachment

[CR35] Mehlich, A. (1984). Mehlich-3 soil test extractant: A modification of the Mehlich-2 extractant. *Communications in Soil Science and Plant Analysis,**15*(12), 1409–1416. 10.1080/00103628409367568

[CR36] Menzies, N. W., Donn, M. J., & Kopittke, P. M. (2007). Evaluation of extractants for estimation of the phytoavailable trace metals in soils. *Environmental Pollution,**145*(1), 121–130. 10.1016/j.envpol.2006.03.021 16777287 10.1016/j.envpol.2006.03.021

[CR37] Mishra, P., Ali, S., Kumar, R., & Shekhar, S. (2025). Global lead contamination in soils, sediments, and aqueous environments: Exposure, toxicity, and remediation. *Journal of Trace Elements and Minerals,**14*, 100259. 10.1016/j.jtemin.2025.100259

[CR38] Nguyen-Phuong, Q., Ponthieu, M., Sayen, S., Marin, B., & Guillon, E. (2024). Adsorption modeling of Cu (II) and Pb (II) onto humin extracted from a peat soil. *Journal of Soils and Sediments,**24*, 769–778. 10.1007/s11368-023-03670-0

[CR39] Ostergren, J. D., BrownJr, G. E. T., Parks, G. A., & Persson, P. (2000). Inorganic ligand effects on Pb (II) sorption to goethite (α-FeOOH). II. Sulfate. *Journal of Colloid and Interface Science,**225*(2), 483–493. 10.1006/jcis.1999.6702 11254288 10.1006/jcis.1999.6702

[CR40] de Paes, É. C., Veloso, G. V., de Castro Filho, M. N., Barroso, S. H., Fernandes-Filho, E. I., Fontes, M. P. F., & Soares, E. M. B. (2023a). Potential of plant species adapted to semi-arid conditions for phytoremediation of contaminated soils. *Journal of Hazardous Materials,**449*, 131034. 10.1016/j.jhazmat.2023.131034 36827724 10.1016/j.jhazmat.2023.131034

[CR41] de Paes, É. C., Veloso, G. V., de Silva, D. L. A., Fernandes-Filho, E. I., Fontes, M. P. F., & Soares, E. M. B. (2023b). Use of modeling to map potentially toxic elements and assess the risk to human health in soils affected by mining activity. *CATENA,**220*, 106662. 10.1016/j.catena.2022.106662

[CR42] Paes, É de. C., de Arruda, D. L., de Lima Camêlo, D., Fernandes, I. O., Veloso, G. V., Ferreira, MdaS., Fontes, M. P. F., Fernandes-Filho, E. I., Santos, M. F. C., & Soares, E. M. B. (2025). The silent risks of urban lead and its impactful consequences in Boquira, Brazil. *Environmental Geochemistry and Health,**47*, 498. 10.1007/s10653-025-02805-1 41087602 10.1007/s10653-025-02805-1

[CR43] Papadopoulos, A. V., Doula, M. K., Zorpas, A. A., Kosmidis, S., Assimakopoulou, A., & Kolovos, C. (2020). Pepper cultivation on a substrate consisting of soil, natural zeolite and olive mill waste sludge: Changes in soil properties. *Comptes Rendus. Chimie, Sustainable Biomass Resources for Environmental, Agronomic, Biomaterials and Energy Applications 1,**23*(11–12), 721–732. 10.5802/crchim.48

[CR44] Porter, S. K., Scheckel, K. G., Impellitteri, C. A., & Ryan, J. A. (2004). Toxic metals in the environment: Thermodynamic considerations for possible immobilization strategies for Pb, Cd, As, and Hg. *Critical Reviews in Environmental Science and Technology,**34*(6), 495–604. 10.1080/10643380490492412

[CR45] Potter, H. A. B., & Yong, R. N. (1999). Influence of iron/aluminium ratio on the retention of lead and copper by amorphous iron–aluminium oxides. *Applied Clay Science,**14*(1–3), 1–26. 10.1016/S0169-1317(98)00045-3

[CR46] Rajendran, S., Priya, T. A. K., Khoo, K. S., Hoang, T. K. A., Ng, H. S., Munawaroh, H. S. H., Karaman, C., Orooji, Y., & Show, P. L. (2022). A critical review on various remediation approaches for heavy metal contaminants removal from contaminated soils. *Chemosphere,**287*, 132369. 10.1016/j.chemosphere.2021.132369 34582930 10.1016/j.chemosphere.2021.132369

[CR47] Rahman, M. A., Shahazi, R., Nova, S. N. B., Uddin, M. R., Hossain, M. S., & Yousuf, A. (2023). Biogas production from anaerobic co-digestion using kitchen waste and poultry manure as substrate: Part 1—Substrate ratio and effect of temperature. *Biomass Conversion and Biorefinery,**13*, 6635–6645. 10.1007/s13399-021-01604-9 34127942 10.1007/s13399-021-01604-9 PMC8189274

[CR48] Rahman, S. U., Qin, A., Zain, M., Mushtaq, Z., Mehmood, F., Riaz, L., Naveed, S., Ansari, M. J., Saeed, M., Ahmad, I., & Shehzad, M. (2024). Pb uptake, accumulation, and translocation in plants: Plant physiological, biochemical, and molecular response—A review. *Heliyon,**10*(6), e27724. 10.1016/j.heliyon.2024.e27724 38500979 10.1016/j.heliyon.2024.e27724 PMC10945279

[CR49] Ruiz, H. A., Ferreira, G. B., & Pereira, J. B. M. (2003). Estimativa da capacidade de campo de Latossolos e Neossolos Quartzarênicos pela determinação do equivalente de umidade. *Revista Brasileira de Ciência do Solo,**27*, 389–393.

[CR78] Santos, H. G. dos, Carvalho Junior, W. de; Dart, R. de O., Áglio, M. L. D., Souza, J. S. de, Pares, J. G., Fontana, A., Martins, A. L. da S. & Oliveira, A. P. de. (2011). O novo mapa de solos do Brasil: legenda atualizada escala 1:5.000.000. Rio de Janeiro, RJ: Embrapa Solos. https://www.infoteca.cnptia.embrapa.br/infoteca/handle/doc/920267

[CR50] Santos, H. G. dos, Jacomine, P. K. T., Anjos, LHCdos., Oliveira, VAde, Lumbreras, J. F., Coelho, M. R., Almeida, JAde, Araujo Filho, JCde, Lima, H. N., Marques, F. A., Oliveira, JBde, & Cunha, T. J. F. (2025). *Sistema Brasileiro de Classificação de Solos*. Brasília, DF: Embrapa Solos. https://www.infoteca.cnptia.embrapa.br/infoteca/handle/doc/1176834

[CR51] Seshadri, B., Bolan, N. S., Choppala, G., Kunhikrishnan, A., Sanderson, P., Wang, H., Currie, L. D., Tsang, D. C. W., Ok, Y. S., & Kim, G. (2017). Potential value of phosphate compounds in enhancing immobilization and reducing bioavailability of mixed heavy metal contaminants in shooting range soil. *Chemosphere,**184*, 197–206. 10.1016/j.chemosphere.2017.05.172 28595145 10.1016/j.chemosphere.2017.05.172

[CR52] Shi, M., Min, X., Ke, Y., Lin, Z., Yang, Z., Wang, S., Peng, N., Yan, X., Luo, S., Wu, J., & Wei, Y. (2021). Recent progress in understanding the mechanism of heavy metals retention by iron (oxyhydr)oxides. *Science of the Total Environment,**752*, 141930. 10.1016/j.scitotenv.2020.141930 32892052 10.1016/j.scitotenv.2020.141930

[CR53] Shuman, L. M. (1979). Zinc, manganese and copper in soil fractions. *Soil Science,**127*(1), 10–17.

[CR55] Silveira, M. L. A., Alleoni, L. R. F., Camargo, O. A., & Casagrande, J. C. (2002). Copper adsorption in oxidic soils after removal of organic matter and iron oxides. *Communications in Soil Science and Plant Analysis,**33*(19–20), 3581–3592. 10.1081/CSS-120015907

[CR56] Sindhu, S. S., Sehrawat, A., & Glick, B. R. (2022). The involvement of organic acids in soil fertility. *Archieves of Microbiology,**204*, 720. 10.1007/s00203-022-03321-x 10.1007/s00203-022-03321-x 36403170

[CR57] Singh, K., Malla, M. A., Kumar, A., & Yadav, S. (2024). Biological monitoring of soil pollution caused by two different zinc species using earthworms. *Environmental Science and Pollution Research,**31*, 57789–57803. 10.1007/s11356-024-34900-8 39292303 10.1007/s11356-024-34900-8

[CR58] Soil Survey Staff. (2022). *Keys to soil taxonomy*. U.S. Department of Agriculture, Natural Resources Conservation Service.

[CR59] Sposito, G., Lund, L. J., & Chang, A. C. (1982). Trace metal chemistry in arid-zone field soils amended with sewage sludge: I. Fractionation of Ni, Cu, Zn, Cd, and Pb in solid phases. *Soil Science Society of America Journal,**46*(2), 260–264. 10.2136/sssaj1982.03615995004600020009x

[CR60] Tavares, S. R. L., & Oliveira, S. A. (2017). Avaliação de diferentes métodos de extração de metais pesados em solos contaminados provenientes de atividades de galvanoplastia. Rio de Janeiro, RJ: Embrapa Solos. https://www.infoteca.cnptia.embrapa.br/infoteca/handle/doc/950815

[CR61] Teixeira, W. G. (2017). Novos métodos de recomendação da necessidade de calagem e especiação de cálcio por espectroscopia XANES em solos de Minas Gerais [Tese, Universidade Federal de Viçosa: Solos e Nutrição de Plantas]. https://locus.ufv.br//handle/123456789/29319

[CR62] Teixeira, P. C., Donagemma, G. K., Fontana, A., & Teixeira, W. G. (Eds.). (2017). *Manual de métodos de análise de solo* (3rd ed.). Brasília, DF: Embrapa Solos. http://www.infoteca.cnptia.embrapa.br/infoteca/handle/doc/1085209

[CR63] Tessier, A., Campbell, P. G. C., & Bisson, M. (1979). Sequential extraction procedure for the speciation of particulate trace metals. *Analytical Chemistry,**51*(7), 844–851. 10.1021/ac50043a017

[CR64] U.S. Environmental Protection Agency. (2007). Guidance for evaluating the oral bioavailability of metals in soils for use in human health risk assessment. *OSWER,**9285*, 7–80.

[CR65] Venegas, A., Rigol, A., & Vidal, M. (2015). Viability of organic wastes and biochars as amendments for the remediation of heavy metal-contaminated soils. *Chemosphere,**119*, 190–198. 10.1016/j.chemosphere.2014.06.009 24995385 10.1016/j.chemosphere.2014.06.009

[CR66] Walker, D. J., Clemente, R., Roig, A., & Bernal, M. P. (2003). The effects of soil amendments on heavy metal bioavailability in two contaminated Mediterranean soils. *Environmental Pollution,**122*(3), 303–312. 10.1016/S0269-7491(02)00287-7 12531318 10.1016/s0269-7491(02)00287-7

[CR67] Walker, D. J., & Bernal, M. P. (2008). The effects of olive mill waste compost and poultry manure on the availability and plant uptake of nutrients in a highly saline soil. *Bioresource Technology,**99*(2), 396–403. 10.1016/j.biortech.2006.12.006 17275292 10.1016/j.biortech.2006.12.006

[CR68] Walkley, A., & Black, I. A. (1934). An examination of the degtjareff method for determining soil organic matter and a proposed modification of the chromic acid titration method. *Soil Science,**37*(1), 29–38. 10.1097/00010694-193401000-00003

[CR69] Xiang, S., Fu, Z., Lu, H., Sun, Y., Shen, Y., & Wu, F. (2025). Antimony and arsenic interactions with iron oxides and aluminum oxides in surface environment: A review focused on processes and mechanisms. *Science of the Total Environment,**979*, 179423. 10.1016/j.scitotenv.2025.179423 40267649 10.1016/j.scitotenv.2025.179423

[CR70] Zhang, Q., & Wang, C. (2020). Natural and human factors affect the distribution of soil heavy metal pollution: A review. *Water, Air, & Soil Pollution,**231*, 350. 10.1007/s11270-020-04728-2

[CR71] Zhou, C., Yuan, H., Ning, C., Li, S., Xia, Z., Zhu, M., & Ma, Q. (2020). Evaluation of different types and amounts of amendments on soil Cd immobilization and its uptake to wheat. *Environmental Management,**65*, 818–828. 10.1007/s00267-020-01287-4 32239252 10.1007/s00267-020-01287-4

[CR72] Zhou, Y., Zhu, Y., & Wong, W. K. (2023). Statistical tests for homogeneity of variance for clinical trials and recommendations. *Contemporary Clinical Trials Communications,**33*, 101119. 10.1016/j.conctc.2023.101119 37143826 10.1016/j.conctc.2023.101119 PMC10151260

[CR73] Zhou, Y., Xie, X., Xia, L., Wang, M., Xiang, J., & Ma, T. (2025). Co-application of organic phosphate fertilizer, manure, and biochar synergistically improves chemical and biological properties of Pb–Zn mine tailings: Insights from a pot trial. *Ecotoxicology and Environmental Safety,**292*, 117984. 10.1016/j.ecoenv.2025.117984 40022826 10.1016/j.ecoenv.2025.117984

[CR74] Zhu, R., Li, M., Ge, F., Xu, Y., Zhu, J., & He, H. (2014). Co-sorption of Cd and phosphate on the surface of a synthetic hydroxyiron–montmorillonite complex. *Clays and Clay Minerals,**62*(2), 79–88. 10.1346/CCMN.2014.0620201

[CR75] Zhu, J., Fu, Q., Qiu, G., Liu, Y., Hu, H., Huang, Q., & Violante, A. (2019). Influence of low molecular weight anionic ligands on the sorption of heavy metals by soil constituents: A review. *Environmental Chemistry Letters,**17*, 1271–1280. 10.1007/s10311-019-00881-1

[CR76] Zorpas, A. A., Navarro Pedreño, J., & Almendro Candel, M. B. (2021). Heavy metal treatment and removal using natural zeolites from sewage sludge, compost, and agricultural soils: A review. *Arabian Journal of Geosciences,**14*, 1098. 10.1007/s12517-021-07443-2

[CR77] Zulfiqar, U., Farooq, M., Hussain, S., Maqsood, M., Hussain, M., Ishfaq, M., Ahmad, M., & Anjum, M. Z. (2019). Lead toxicity in plants: Impacts and remediation. *Journal of Environmental Management,**250*, 109557. 10.1016/j.jenvman.2019.109557 31545179 10.1016/j.jenvman.2019.109557

