# Supplementary material for: Lead fractionation and availability in contaminated soils: a comparative study of organic wastes and zeolite as stabilizing agents
Source: Environ Geochem Health. 2026 Aug 5;48(12):504. doi: 10.1007/s10653-026-03373-8 (PMC13442485; doi:10.1007/s10653-026-03373-8)
Supplement: Supplementary file 1 [file 10653_2026_3373_MOESM1_ESM.docx]

**Lead fractionation and bioavailability in contaminated soils: A comparative study of organic wastes and zeolite as stabilizing agents**

Priscila Soares de Resende^a^, Gordon W Price^b^, Rafael da Silva Teixeira^a^ , Isabela C. F. Vasques^a^*

^a^ Department of Soils, Federal University of Viçosa, Viçosa, Minas Gerais 36570-900, Brazil

^b^ Faculty of Agriculture, Dalhousie University, PO Box 550, 21 Cox Rd., Truro, NS B2N 5E3, Canada

*Corresponding author: isabela.filardi@ufv.br

Orcid ID: 0000-0002-4798-9384

**Supplementary material**

Sewage Sludge

pH

|  | CK | SS 1% | SS 5% | SS 10% |
| --- | --- | --- | --- | --- |
| 1 d | 6.6 | 6.26667 | 5.5 | 5.13333 |
| 3 d | 6.83333 | 6.6 | 6.23333 | 6.06667 |
| 5 d | 6.4 | 6.53333 | 6.1 | 6.23333 |
| 8 d | 7.3 | 7.13333 | 6.8 | 6.56667 |
| 15 d | 7.06667 | 7.03333 | 6.76667 | 6.66667 |
| 21 d | 7.13333 | 7.13333 | 6.93333 | 7 |
| 30 d | 7.13333 | 7.1 | 6.96667 | 6.73333 |

Cattle Manure

pH

|  | CK | CM 1% | CM 5% | CM 10% |
| --- | --- | --- | --- | --- |
| 1 d | 6.6 | 6.8 | 6.86667 | 6.86667 |
| 3 d | 6.83333 | 6.83333 | 6.86667 | 6.9 |
| 5 d | 6.4 | 6.73333 | 6.76667 | 6.83333 |
| 8 d | 7.3 | 7.2 | 7.16667 | 7.1 |
| 15 d | 7.06667 | 7.16667 | 7 | 7.16667 |
| 21 d | 7.13333 | 7.3 | 7.3 | 7.1 |
| 30 d | 7.13333 | 7.33333 | 7.2 | 7.1 |

Poultry Manure

pH

|  | CK | PM 1% | PM 5% | PM 10% |
| --- | --- | --- | --- | --- |
| 1 d | 6.6 | 6.96667 | 7.36667 | 7.56667 |
| 3 d | 6.83333 | 7.03333 | 7.26667 | 7.53333 |
| 5 d | 6.4 | 6.93333 | 7.23333 | 7.53333 |
| 8 d | 7.3 | 7.16667 | 7.3 | 7.26667 |
| 15 d | 7.06667 | 7.3 | 7.06667 | 7.33333 |
| 21 d | 7.13333 | 6.96667 | 6.9 | 7.06667 |
| 30 d | 7.13333 | 7.23333 | 7.13333 | 7.33333 |

**Fig. S1** Variation in soil pH over a 30-day incubation with different organic amendments: (a) sewage sludge (SS), (b) cattle manure (CM), and (c) poultry manure (PM), applied at rates of 1%, 5%, and 10% (v/v), compared with the control (CK; bare soil + mine tailing). Values represent means ± standard error (n = 3)

Fine zeolite

pH

|  | CK | <0.4mm 1% | <0.4mm5% | <0.4mm10% |
| --- | --- | --- | --- | --- |
| 1 d | 6.6 | 6.7 | 6.8 | 6.93333 |
| 3 d | 6.76667 | 6.73333 | 7.13333 | 7.06667 |
| 5 d | 6.43333 | 6.73333 | 6.56667 | 6.63333 |
| 8 d | 7.33333 | 6.93333 | 7.06667 | 7.16667 |
| 15 d | 7.1 | 7.1 | 7.3 | 7.2 |
| 21 d | 7.1 | 7.2 | 7.4 | 7.3 |
| 30 d | 7.06667 | 7.26667 | 7.2 | 7.26667 |

Medium zeolite

pH

|  | CK | 2-8mm 1% | 2-8mm 5% | 2-8mm 10% |
| --- | --- | --- | --- | --- |
| 1 d | 6.6 | 6.6 | 6.46667 | 6.36667 |
| 3 d | 6.76667 | 6.7 | 6.73333 | 6.83333 |
| 5 d | 6.43333 | 6.3 | 6.3 | 6.6 |
| 8 d | 7.33333 | 7.26667 | 7.1 | 7 |
| 15 d | 7.1 | 7.06667 | 7.03333 | 6.93333 |
| 21 d | 7.1 | 7.13333 | 7.23333 | 7.2 |
| 30 d | 7.06667 | 7.16667 | 7.2 | 7.2 |

Coarse zeolite

pH

|  | CK | >8mm 1% | >8mm 5% | >8mm 10% |
| --- | --- | --- | --- | --- |
| 1 d | 6.6 | 6.83333 | 6.53333 | 6.83333 |
| 3 d | 6.76667 | 7.1 | 7.2 | 7.06667 |
| 5 d | 6.43333 | 6.53333 | 6.43333 | 6.6 |
| 8 d | 7.33333 | 7.23333 | 7.1 | 7.3 |
| 15 d | 7.1 | 7.2 | 7.13333 | 7.26667 |
| 21 d | 7.1 | 7.23333 | 7.33333 | 7.46667 |
| 30 d | 7.06667 | 7.2 | 7.3 | 7.4 |

**Fig. S2** Variation in soil pH over a 30-day incubation with different particle size of zeolite: (a) fine zeolite (< 0.4 mm), (b) medium zeolite (2 – 8 mm) and (c) coarse zeolite (> 8 mm), applied at rates of 1%, 5%, and 10% (v/v), compared with the control (CK: bare soil + mine tailing). Values are means ± standard error (n = 3)

**Table S1**. Variation in soil pH and acid-labile Pb concentration over a 30-day incubation with sewage sludge (SS) applied at rates of 1%, 5%, and 10% (v/v), compared with the control (CK; bare soil + mine tailing)

|  | pH | | | | | | | Acid-labile Pb | | | | | | |
| --- | --- | --- | --- | --- | --- | --- | --- | --- | --- | --- | --- | --- | --- | --- |
|  | 1d | 3d | 5d | 8d | 15d | 21d | 30d | 1d | 3d | 5d | 8d | 15d | 21d | 30d |
| CK | 6.6Aa | 6.8Aa | 6.4Ba | 7.3Ca | 7.1Ca | 7.1Ca | 7.1Ca | 188Aab | 185Aa | 257Ba | 226Ba | 225Ba | 194Bb | 230Ba |
| SS1% | 6.3Aa | 6.6Ba | 6.5Ba | 7.1Cab | 7.0Ca | 7.1Ca | 7.1Ca | 215Aa | 208Aa | 267Ba | 208Cab | 257Db | 234Da | 233Da |
| SS5% | 5.5Ab | 6.2Bb | 6.1Ba | 6.8Cbc | 6.8Ca | 6.9Ca | 7.0Ca | 161Ab | 183Ba | 232Ca | 199Cab | 215Ca | 181Cb | 210Ca |
| SS10% | 5.1Ab | 6.1Bb | 6.2Ba | 6.6Cc | 6.7Ca | 7.0Ca | 6.7Ca | 167Aab | 190Aa | 243Ba | 181Cb | 201Ca | 192Cb | 230Da |

In rows, comparisons were performed between consecutive sampling times (Day 3 vs. Day 1; Day 5 vs. Day 3; Day 8 vs. Day 5; Day 15 vs. Day 8; Day 21 vs. Day 15; and Day 30 vs. Day 21). Different uppercase letters within rows indicates significant differences based on Holm-Bonferroni adjusted contrasts (*p* < 0.05). In columns, comparisons were performed among all pairwise group means. Different lowercase letters within columns indicate significant differences according to Tukey’s test (p < 0.05). Values are means ± standard error, n = 3.

**Table S2.** Variation in soil pH and acid-labile Pb concentration over a 30-day incubation with cattle manure (CM) applied at rates of 1%, 5%, and 10% (v/v), compared with the control (CK; bare soil + mine tailing).

|  | pH | | | | | | | Acid-labile Pb | | | | | | |
| --- | --- | --- | --- | --- | --- | --- | --- | --- | --- | --- | --- | --- | --- | --- |
|  | 1d | 3d | 5d | 8d | 15d | 21d | 30d | 1d | 3d | 5d | 8d | 15d | 21d | 30d |
| CK | 6.6Aa | 6.8Aa | 6.4Bb | 7.3Ca | 7.1Ca | 7.1Ca | 7.1Ca | 188Aa | 185Aa | 257Ba | 226Ba | 225Ba | 194Ba | 230Ba |
| CM1% | 6.8Aa | 6.8Aa | 6.7Aa | 7.2Ba | 7.2Ba | 7.3Ba | 7.3Ba | 213Aa | 172Aa | 279Ba | 201Cb | 236Ca | 209Ca | 208Ca |
| CM5% | 6.9Aa | 6.9Aa | 6.8Aa | 7.0Ba | 7.0Ba | 7.3Ba | 7.2Ba | 181Aa | 191Aa | 287Ba | 215Ca | 220Ca | 214Ca | 211Ca |
| CM10% | 6.9Aa | 6.9Aa | 6.8Aa | 7.1Ba | 7.2Ba | 7.1Ba | 7.1Ba | 184Aa | 176Aa | 262Ba | 184Cb | 180Cb | 220Ca | 208Ca |

In rows, comparisons were performed between consecutive sampling times (Day 3 vs. Day 1; Day 5 vs. Day 3; Day 8 vs. Day 5; Day 15 vs. Day 8; Day 21 vs. Day 15; and Day 30 vs. Day 21). Different uppercase letters within rows indicates significant differences based on Holm-Bonferroni adjusted contrasts (*p* < 0.05). In columns, comparisons were performed among all pairwise group means. Different lowercase letters within columns indicate significant differences according to Tukey’s test (p < 0.05). Values are means ± standard error, n = 3.

**Table S3.** Variation in soil pH and acid-labile Pb concentration over a 30-day incubation with poultry manure (PM) applied at rates of 1%, 5%, and 10% (v/v), compared with the control (CK; bare soil + mine tailing).

|  | pH | | | | | | | Acid-labile Pb | | | | | | |
| --- | --- | --- | --- | --- | --- | --- | --- | --- | --- | --- | --- | --- | --- | --- |
|  | 1d | 3d | 5d | 8d | 15d | 21d | 30d | 1d | 3d | 5d | 8d | 15d | 21d | 30d |
| CK | 6.6Ac | 6.8Ac | 6.4Bd | 7.3Ca | 7.1Ca | 7.1Ca | 7.1Ca | 188Aab | 185Aa | 257Ba | 226Ba | 225Ba | 194Ba | 230Ba |
| PM1% | 7.0Ab | 7.0Abc | 6.9Ac | 7.2Aa | 7.3Aa | 7.0Ba | 7.2Aa | 202Aa | 183Aa | 226Aab | 191Ab | 196Aa | 190Aa | 235Aa |
| PM5% | 7.4Aa | 7.3Ab | 7.2Ab | 7.3Aa | 7.1Aa | 6.9Aa | 7.1Aa | 169Aab | 146Bab | 220Cab | 172Dbc | 150Db | 164Dab | 190Db |
| PM10% | 7.6Aa | 7.5Aa | 7.5Aa | 7.3Ba | 7.3Ba | 7.1Ca | 7.3Da | 148Ab | 114Bb | 184Cb | 154Dc | 144Db | 144Db | 156Dc |

In rows, comparisons were performed between consecutive sampling times (Day 3 vs. Day 1; Day 5 vs. Day 3; Day 8 vs. Day 5; Day 15 vs. Day 8; Day 21 vs. Day 15; and Day 30 vs. Day 21). Different uppercase letters within rows indicates significant differences based on Holm-Bonferroni adjusted contrasts (*p* < 0.05). In columns, comparisons were performed among all pairwise group means. Different lowercase letters within columns indicate significant differences according to Tukey’s test (p < 0.05). Values are means ± standard error, n = 3.

**Table S4.** Distribution of the Pb concentration among the sequential extraction for the organic amendments experiment

|  | mg Pb/kg soil | | | | | | |
| --- | --- | --- | --- | --- | --- | --- | --- |
|  | F1 | F2 | F3 | F4 | F5 | F6 | F7 |
| CK | 0.00 | 0.00 | 379.53 | 1.48 | 195.09 | 12.36 | 486.70 |
|  | 0.00 | 0.00 | 375.22 | 1.19 | 172.90 | 15.54 | 399.50 |
|  | 0.00 | 0.00 | 351.29 | 1.63 | 213.32 | 13.22 | 510.30 |
| SS 1% | 0.00 | 0.70 | 433.13 | 1.39 | 212.17 | 10.56 | 563.40 |
|  | 0.00 | 0.51 | 386.42 | 1.75 | 206.56 | 7.34 | 427.60 |
|  | 0.00 | 0.38 | 408.19 | 2.39 | 222.46 | 5.27 | 477.90 |
| SS 5% | 0.00 | 0.41 | 361.79 | 3.75 | 203.44 | 11.66 | 434.80 |
|  | 0.00 | 0.53 | 365.52 | 4.23 | 182.14 | 15.66 | 493.00 |
|  | 0.00 | 0.23 | 374.26 | 3.07 | 155.34 | 15.44 | 443.90 |
| SS 10% | 0.00 | 0.67 | 356.29 | 1.87 | 205.37 | 15.98 | 465.50 |
|  | 0.00 | 0.93 | 326.39 | 2.11 | 200.57 | 12.45 | 497.90 |
|  | 0.00 | 0.72 | 370.87 | 1.20 | 188.63 | 16.30 | 512.20 |
| CM 1% | 0.00 | 0.41 | 445.18 | 1.59 | 198.73 | 11.50 | 427.50 |
|  | 0.00 | 0.46 | 448.31 | 1.91 | 201.34 | 13.03 | 474.50 |
|  | 0.00 | 0.13 | 373.94 | 1.04 | 169.20 | 9.84 | 440.40 |
| CM 5% | 0.00 | 0.35 | 411.29 | 1.97 | 168.97 | 8.60 | 450.30 |
|  | 0.00 | 0.31 | 437.50 | 1.54 | 199.65 | 9.78 | 506.10 |
|  | 0.00 | 0.21 | 352.92 | 1.29 | 187.62 | 6.89 | 468.20 |
| CM 10% | 0.00 | 0.52 | 570.58 | 1.42 | 237.15 | 10.80 | 546.70 |
|  | 0.00 | 0.63 | 718.44 | 1.15 | 191.89 | 9.51 | 445.60 |
|  | 0.00 | 0.73 | 608.65 | 1.68 | 202.23 | 12.41 | 504.70 |
| PM 1% | 0.00 | 0.68 | 349.25 | 3.11 | 154.70 | 15.76 | 490.30 |
|  | 0.00 | 1.71 | 328.74 | 3.59 | 162.61 | 13.33 | 508.20 |
|  | 0.00 | 1.08 | 309.64 | 3.82 | 150.11 | 14.12 | 500.00 |
| PM 5% | 0.00 | 0.14 | 328.98 | 5.87 | 162.68 | 17.86 | 534.10 |
|  | 0.00 | 0.35 | 254.03 | 5.10 | 168.81 | 20.23 | 592.90 |
|  | 0.00 | 0.21 | 211.19 | 4.71 | 168.84 | 16.12 | 463.20 |
| PM 10% | 0.08 | 1.56 | 173.22 | 5.64 | 131.57 | 27.24 | 526.60 |
|  | 0.13 | 1.79 | 182.07 | 6.39 | 158.21 | 24.48 | 510.70 |
|  | 0.10 | 1.97 | 192.95 | 5.77 | 147.82 | 31.12 | 576.30 |

**Table S5.** Variation in soil pH and acid-labile Pb concentration over a 30-day incubation with fine zeolite (<0.4 mm) applied at rates of 1%, 5%, and 10% (v/v), compared with the control (CK; bare soil + mine tailing).

|  | pH | | | | | | | Acid-labile Pb | | | | | | |
| --- | --- | --- | --- | --- | --- | --- | --- | --- | --- | --- | --- | --- | --- | --- |
|  | 1d | 3d | 5d | 8d | 15d | 21d | 30d | 1d | 3d | 5d | 8d | 15d | 21d | 30d |
| CK | 6.6Aa | 6.8Aa | 6.4Aa | 7.3Ba | 7.1Ba | 7.1Bb | 7.1Ba | 180Aa | 177Aa | 253Ba | 249Ba | 230Ba | 208a | 229Ba |
| <0.4mm1% | 6.7Abc | 6.7Aa | 6.7Aa | 6.9Ab | 7.1Aa | 7.2Aab | 7.3Aa | 121Ab | 134Ab | 227Ba | 155Cb | 171Cb | 168Cb | 177Cb |
| <0.4mm5% | 6.8Aab | 7.1Ba | 6.6Ca | 7.1Dab | 7.3Da | 7.4Da | 7.2Da | 90Ac | 100Ac | 146Bb | 114Cc | 120Cc | 131Cc | 137Cc |
| <0.4mm10% | 6.9Aa | 7.1Aa | 6.6Ba | 7.2Cab | 7.2Ca | 7.3Cab | 7.3Ca | 55Ad | 70Ad | 118Bb | 88Bc | 88Bc | 91Bd | 90Bd |

In rows, comparisons were performed between consecutive sampling times (Day 3 vs. Day 1; Day 5 vs. Day 3; Day 8 vs. Day 5; Day 15 vs. Day 8; Day 21 vs. Day 15; and Day 30 vs. Day 21). Different uppercase letters within rows indicates significant differences based on Holm-Bonferroni adjusted contrasts (*p* < 0.05). In columns, comparisons were performed among all pairwise group means. Different lowercase letters within columns indicate significant differences according to Tukey’s test (p < 0.05). Values are means ± standard error, n = 3.

**Table S6.** Variation in soil pH and acid-labile Pb concentration over a 30-day incubation with medium zeolite (2-8 mm) applied at rates of 1%, 5%, and 10% (v/v), compared with the control (CK; bare soil + mine tailing).

|  | pH | | | | | | | Acid-labile Pb | | | | | | |
| --- | --- | --- | --- | --- | --- | --- | --- | --- | --- | --- | --- | --- | --- | --- |
|  | 1d | 3d | 5d | 8d | 15d | 21d | 30d | 1d | 3d | 5d | 8d | 15d | 21d | 30d |
| CK | 6.6Aa | 6.8Aa | 6.4Aa | 7.3Ba | 7.1Ba | 7.1Ba | 7.1Ba | 180Aa | 177Aa | 253Ba | 249Ba | 230Ba | 208a | 229Ba |
| 2-8mm1% | 6.6Aa | 6.7Aa | 6.3Ba | 7.3Cab | 7.1Ca | 7.1Ca | 7.2Ca | 133Ab | 152Aab | 185Ab | 183Ab | 179Ab | 171Ab | 185Ab |
| 2-8mm5% | 6.5Aa | 6.7Aa | 6.3Ba | 7.1Cbc | 7.0Ca | 7.2Ca | 7.2Ca | 126Ab | 137Ab | 148Ac | 157Ab | 163Ab | 151Ab | 159Ab |
| 2-8mm10% | 6.4Aa | 6.8Aa | 6.6Aa | 7.0Bc | 6.9Ba | 7.2Ba | 7.2Ba | 86Ac | 114Ac | 187Bb | 122Cc | 118Cc | 138Cb | 149Cb |

In rows, comparisons were performed between consecutive sampling times (Day 3 vs. Day 1; Day 5 vs. Day 3; Day 8 vs. Day 5; Day 15 vs. Day 8; Day 21 vs. Day 15; and Day 30 vs. Day 21). Different uppercase letters within rows indicates significant differences based on Holm-Bonferroni adjusted contrasts (*p* < 0.05). In columns, comparisons were performed among all pairwise group means. Different lowercase letters within columns indicate significant differences according to Tukey’s test (p < 0.05). Values are means ± standard error, n = 3.

**Table S7.** Variation in soil pH and acid-labile Pb concentration over a 30-day incubation with coarse zeolite (>8 mm) applied at rates of 1%, 5%, and 10% (v/v), compared with the control (CK; bare soil + mine tailing).

|  | pH | | | | | | | Acid-labile Pb | | | | | | |
| --- | --- | --- | --- | --- | --- | --- | --- | --- | --- | --- | --- | --- | --- | --- |
|  | 1d | 3d | 5d | 8d | 15d | 21d | 30d | 1d | 3d | 5d | 8d | 15d | 21d | 30d |
| CK | 6.6Aa | 6.8Ab | 6.4Aa | 7.3Ba | 7.1Ba | 7.1Bb | 7.1Bb | 180Aa | 177Aa | 253Ba | 249Ba | 230Ba | 208a | 229Ba |
| >8mm1% | 6.8Aa | 7.1Aa | 6.5Aa | 7.2Ba | 7.2Ba | 7.2Bb | 7.2Bab | 165Aa | 180Aa | 250Ba | 204Bab | 187Bbc | 204Ba | 200Bb |
| >8mm5% | 6.5Aa | 7.2Ba | 6.4Ca | 7.1Da | 7.1Da | 7.3Dab | 7.3Dab | 147Aa | 157Ab | 213Bab | 200Bab | 228Bab | 175Cab | 200Cb |
| >8mm10% | 6.8Aa | 7.1Aa | 6.6Ba | 7.3Ca | 7.3Ca | 7.5Ca | 7.4Ca | 153Aa | 160Aab | 184Ab | 171Ab | 175Ac | 154Ab | 179Ab |

In rows, comparisons were performed between consecutive sampling times (Day 3 vs. Day 1; Day 5 vs. Day 3; Day 8 vs. Day 5; Day 15 vs. Day 8; Day 21 vs. Day 15; and Day 30 vs. Day 21). Different uppercase letters within rows indicates significant differences based on Holm-Bonferroni adjusted contrasts (*p* < 0.05). In columns, comparisons were performed among all pairwise group means. Different lowercase letters within columns indicate significant differences according to Tukey’s test (p < 0.05). Values are means ± standard error, n = 3.

**Table S8.** Distribution of the Pb concentration among the sequential extraction for the zeolite experiment

|  | mg Pb/kg soil | | | | | | |
| --- | --- | --- | --- | --- | --- | --- | --- |
|  | F1 | F2 | F3 | F4 | F5 | F6 | F7 |
| CK | 0.00 | 0.17 | 424.84 | 1.66 | 205.29 | 9.51 | 565.10 |
|  | 0.00 | 0.20 | 421.44 | 2.08 | 239.68 | 9.58 | 451.90 |
|  | 0.00 | 0.10 | 375.63 | 1.29 | 202.83 | 8.33 | 449.80 |
| Z <0.4mm 1% | 0.00 | 0.45 | 455.06 | 2.37 | 206.25 | 12.55 | 452.60 |
|  | 0.00 | 0.32 | 465.13 | 2.32 | 202.82 | 11.05 | 341.20 |
|  | 0.00 | 0.35 | 478.54 | 2.74 | 212.67 | 17.20 | 445.70 |
| Z <0.4mm 5% | 0.00 | 0.44 | 452.86 | 2.48 | 168.31 | 15.03 | 518.40 |
|  | 0.00 | 0.55 | 428.70 | 2.21 | 142.58 | 17.40 | 598.10 |
|  | 0.00 | 0.53 | 481.64 | 2.38 | 188.58 | 20.22 | 584.50 |
| Z <0.4mm 10% | 0.00 | 0.51 | 414.58 | 2.23 | 144.24 | 21.07 | 564.50 |
|  | 0.00 | 0.64 | 423.38 | 1.79 | 153.07 | 23.31 | 467.50 |
|  | 0.00 | 0.49 | 449.27 | 1.87 | 149.45 | 24.13 | 451.50 |
| Z 2-8mm 1% | 0.00 | 0.10 | 483.51 | 1.80 | 243.62 | 14.70 | 550.00 |
|  | 0.00 | 0.12 | 426.58 | 2.14 | 203.54 | 12.93 | 522.10 |
|  | 0.00 | 0.18 | 548.18 | 2.06 | 259.38 | 18.07 | 622.80 |
| Z 2-8mm 5% | 0.00 | 0.18 | 505.29 | 2.18 | 203.36 | 22.01 | 491.40 |
|  | 0.00 | 0.12 | 458.41 | 2.33 | 198.48 | 18.59 | 622.60 |
|  | 0.00 | 0.10 | 389.98 | 1.66 | 149.89 | 17.61 | 531.90 |
| Z 2-8mm 10% | 0.00 | 0.10 | 326.76 | 1.76 | 162.82 | 13.70 | 409.70 |
|  | 0.00 | 0.15 | 333.88 | 2.09 | 119.87 | 12.11 | 429.50 |
|  | 0.00 | 0.17 | 393.51 | 1.77 | 159.57 | 11.03 | 429.70 |
| Z > 8mm 1% | 0.00 | 0.00 | 428.54 | 1.23 | 202.17 | 12.49 | 456.00 |
|  | 0.00 | 0.00 | 391.18 | 0.79 | 165.19 | 12.15 | 474.90 |
|  | 0.00 | 0.00 | 453.44 | 1.70 | 217.17 | 11.07 | 544.40 |
| Z > 8mm 5% | 0.00 | 0.00 | 498.85 | 3.05 | 189.67 | 16.20 | 570.10 |
|  | 0.00 | 0.00 | 408.95 | 2.13 | 224.26 | 14.62 | 663.10 |
|  | 0.00 | 0.00 | 513.14 | 3.07 | 234.92 | 13.18 | 571.10 |
| Z > 8mm 10% | 0.00 | 0.00 | 413.36 | 2.75 | 164.85 | 13.05 | 552.90 |
|  | 0.00 | 0.00 | 328.05 | 1.89 | 119.31 | 15.13 | 457.90 |
|  | 0.00 | 0.00 | 389.87 | 2.37 | 138.21 | 11.53 | 598.30 |
